# Linking anatomical and histological traits of the digestive tract to resource consumption and assimilation of omnivorous tetra fishes

**DOI:** 10.1002/ece3.11375

**Published:** 2024-05-02

**Authors:** Mayara Pereira Neves, João Paulo de Arruda Amorim, Rosilene Luciana Delariva, Pavel Kratina, Clarice Bernhardt Fialho

**Affiliations:** ^1^ Department of Biosciences Rice University Houston Texas USA; ^2^ Programa de Pós‐Graduação em Biologia Animal, Departamento de Zoologia, Instituto de Biociências Universidade Federal do Rio Grande do Sul Porto Alegre RS Brazil; ^3^ Laboratório de Biologia Tecidual e da Reprodução Universidade Estadual do Oeste do Paraná, Centro de Ciências Biológicas e da Saúde Cascavel PR Brazil; ^4^ Laboratório de Ictiologia, Ecologia e Biomonitoramento Universidade Estadual do Oeste do Paraná, Centro de Ciências Biológicas e da Saúde Cascavel PR Brazil; ^5^ School of Biological and Behavioural Sciences Queen Mary University of London London UK

**Keywords:** animal and plant diet, Characidae, histology, intestine, morphology, omnivory

## Abstract

This study explores the interplay between digestive tract traits, food intake, and assimilation in omnivorous tetra fishes (*Psalidodon bifasciatus*, *P.* aff. *gymnodontus*, and *Bryconamericus ikaa*) from the Iguaçu River basin, an ecologically significant region known for high endemism. We hypothesize that variations in digestive tracts across species would be associated with differences in diet, isotopic composition in fish tissues, and overall diet assimilation. To test this, we employed stereoscopic and light microscopy to characterize the gross anatomy, histomorphology, and histochemistry of fish digestive tracts. Additionally, we used stomach content and stable isotope analyses to trace fish diets. While these tetra fishes shared histological structures, disparities were noted in anatomical digestive traits and diet preferences. The smallest species, *B. ikaa*, with a shorter intestine, had fewer pyloric caeca and primarily consumed animal‐based diets. Conversely, *P. bifasciatus* and *P.* aff. *gymnodontus*, with longer intestines, displayed numerous pyloric caeca and consumed a balanced mix of animal and plant items. Despite anatomical and dietary differences, all three species predominantly assimilated animal‐origin food. The tetra fishes had histological variations among digestive tract segments, with the esophagus having the thickest muscular layer, gradually thinning towards the posterior intestine. The final portion of the intestine exhibited a significant expansion in the lumen perimeter, while the esophagus had the smallest lumen area. Goblet cells were most concentrated in the posterior intestine for all species. The gross anatomy of these tetra fishes aligns with their omnivorous habit, while diet assimilation was dominated by animal‐origin food. These findings provide crucial insights into the structural and tissue characteristics of their digestive systems, laying the groundwork for deeper exploration into the physiological aspects of their digestive tracts and enhancing our understanding of their feeding strategies.

## Graphical abstract

This study investigates digestive traits and dietary patterns in omnivorous tetra fishes from the Iguaçu River basin, known for high endemism. The researchers found that while the tetra fishes shared histological structures, anatomical differences in digestive traits correlated with distinct dietary preferences. Despite variations in gross anatomy and food intake, all species predominantly assimilated food of animal origin, providing fundamental insights into the structural and tissue characteristics of their digestive systems.

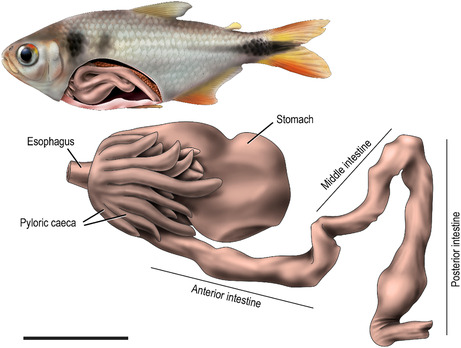

## 1 INTRODUCTION

Resource acquisition is vital for the survival of species, with animals evolving to exploit various food types (Karasov et al., 2011). Wild fishes face the challenge of meeting their nutritional needs amidst variations in food availability and quality, possibly driving the development of diverse digestive adaptations. The variability in the digestive system appears to correlate with materials resistant to digestion (Karasov et al., 2011). Physical, chemical, and enzymatic processes contribute to digestion, while absorption and assimilation incorporate nutrients into cells and tissues for various physiological functions (Bakke et al., 2010; Nielsen et al., 2018). Consequently, understanding the morphological traits of digestive systems is crucial for comprehending the relationship between ingestion, digestion, absorption, and assimilation of food, particularly in dynamic environments like tropical streams and rivers (Delariva & Neves, 2020; Neves et al., 2023; Wilson & Castro, 2010).

The morphology of the digestive tract in teleost fishes, essential for their diverse feeding strategies, has been extensively studied, revealing various anatomical adaptations and trophic position (Moraes & Almeida, 2020). However, our understanding of the diversity of teleost digestive systems remains limited due to the highly morphological and phylogenetic diversification (Albrecht et al., 2001; Dzhumaliyev, 1982; Fanta et al., 2001; Karasov et al., 2011). While histological relationships between diet and digestive tract have previously been documented, descriptive data, especially for small omnivorous Neotropical fishes, are still lacking (Alonso et al., 2015; Cardoso et al., 2015; Rincón et al., 2023). Teleosts exhibit morphological adaptations reflecting their varied diets, influencing the length of their intestines and nutrient acquisition efficiency (Ghilardi et al., 2021; Ribble & Smith, 1983; Wilson & Castro, 2010). Studies of digestive systems advance our understanding of fish physiology and feeding habits (Fugi & Hahn, 1991; Végaz‐Velez, 1972), crucial for comprehending digestive performance and evolution, especially in species‐rich yet phylogenetically correlated neotropical environments (Albert et al., 2020; Baumgartner et al., 2012).

The family Characidae is known for its remarkable diversity among fish taxa in the Neotropical freshwater ecosystems (Fricke et al., 2020). The small characids, commonly referred to as tetra fish, are particularly abundant in rivers, lakes, and streams. *Psalidodon*, *Astyanax*, and *Bryconamericus* are the most species‐rich genera of the Characidae family. Omnivorous diet and trophic plasticity have been well documented for these genera that often consume both animal and plant food items (Bonato et al., 2018; Neves et al., 2023; Pini et al., 2019). These species are often opportunistic feeders with the ability to change their diet across seasons and space depending on food resource availability (Neves et al., 2021, 2023; Pini et al., 2019; Quirino et al., 2015). Although the trophic ecology of these species has been extensively studied (Bonato et al., 2018; Delariva & Neves, 2020; Neves et al., 2023; Pini et al., 2019), we still know little about the histological characteristics of their digestive tract and how these traits are related to food ingestion, digestion, absorption, and assimilation. Moreover, recent stable isotope analyses indicate that while tetra fishes readily ingest plant food items, they rarely assimilate or incorporate these types of food into their tissues (Bonato et al., 2018; Neves et al., 2021, 2023). This finding raises questions about the role of plant material in the diets of tetra fishes.

In this study, we explored the relationships among morphological characteristics of the digestive tract, food intake, isotopic composition, and resource assimilation of three native omnivorous fish species—*Psalidodon bifasciatus* (Garavello & Sampaio 2010), *Psalidodon* aff. *gymnodontus* Eigenmann 1911, and *Bryconamericus ikaa* Casciotta, Almirón & Azpelicueta 2004—from headwater streams in the Lower Iguaçu River basin (= Iguassu), Southern Brazil. The Iguaçu River basin stands out as an ecological region renowned for its uniqueness biodiversity, high endemism, and pivotal role in supporting various ecosystem types. Notably, *B. ikaa* and *P*. aff. *gymnodontus* are endemic to the Iguaçu river basin, underlining the importance of understanding their natural history in light of increasing anthropogenic pressures (Neves et al., 2023). Consequently, our study aimed to accomplish the following objectives: (i) describe the anatomical, histological, and histochemical traits of the digestive tract and diet of these small tetra fishes; (ii) investigate the potential relationship between intestine length, diet, and isotopic composition given their omnivorous feeding habit; and (iii) test the hypothesis that variations in the digestive tract among the species correspond to differences in resource utilization (animal and plant food items), isotopic composition, and assimilation. Such evidence would advance our understanding of morphology, trophic plasticity, and species coexistence among fish species in the Iguassu Ecoregion, offering crucial ecological insights. Understanding these relationships is essential to improve conservation efforts, ensuring the preservation of the unique biodiversity and ecological integrity of this ecoregion.

## 2 MATERIALS AND METHODS

### 2.1 Species acquisition

To collect the tetra fishes, we sampled two headwater streams (S1: 25°9′10.25″ S, 53°16′41.86″ W; S2: 25°6′7.17″ S, 53°18′42.25″ W) in July and December 2017 using electrofishing with three passes of 40 min. During each fieldwork we anesthetized the specimens in eugenol (Eugenol, 2 drops per litter; American Veterinary Medical Association, 2001; Javahery et al., 2012). Afterwards, we identified specimens according to specific identification keys (Baumgartner et al., 2012; Ota et al., 2018), measured the total and standard length from the left side of the individuals using a digital caliper (accuracy of 0.01 mm), and weight (g) using a portable digital scale (Vazzoler, 1996). We then visually inspected the presence of mature gonads of all sampled fishes and selected adult specimens of each species for further analysis (Vazzoler, 1996).

For the histological analysis, during each sampling period, we selected five individuals of each species from each stream. We dissected their digestive tracts with a longitudinal incision along the ventral region. Then, we fixed the digestive tract in ALFAC (alcohol 80%, 85 mL; formaldehyde PA, 10 mL; and acetic acid PA, 5 mL; Caputo et al., 2011) and conserved in 70% alcohol. We also fixed additional specimens in formaldehyde 10% and conserved them in 70% alcohol for stomach content analysis. Finally, for stable isotope analysis, in the summer (December 2017), we dissected samples of dorsal muscle tissue from 10 to 15 adult specimens per species and stream. Following the methodology from Neves et al. (2021, 2023), we manually sampled basal resources (terrestrial plants and sedimentary organic matter—SOM) and putative prey (aquatic and terrestrial invertebrates). We immediately stored all samples on ice for further processing in the laboratory.

We collected the fishes under authorization from the Instituto Chico Mendes de Conservação da Biodiversidade (ICMBio) (license number 25039). The project was ethically and methodologically approved by the Ethics Committee on Animal Use of the Universidade Federal do Rio Grande do Sul (CEUA – 32734). To comply with their protocols for fish usage, we deposited the voucher specimens in the fish collection of the Departamento de Zoologia at the Universidade Federal do Rio Grande do Sul (*P. bifasciatus* UFRGS 26235; *P*. aff. *gymnodontus* UFRGS 25725; *B. ikaa* UFRGS 26246).

### 2.2 Stomach content analysis

To estimate stomach contents, we analyzed 244 adult fish specimens (*B. ikaa*: 31; *P*. aff. *gymnodontus*: 96; *P. bifasciatus*: 117). We carefully extracted the stomachs of the fishes under optical and stereoscopic microscopes (Opton TIM‐2B WF10X) to identify the stomach contents with the highest possible taxonomic precision. For the identification of algae we used specialized literature by Bicudo and Bicudo (1970), while for invertebrates, we used Mugnai et al. (2010). We applied the volumetric method to quantify the food items (Hyslop, 1980) with graduated test tubes and glass counting plates (Hellawell & Abel, 1971).

### 2.3 Stable isotope analyses

For stable isotope analysis, we examined a total of 55 fish specimens (*B. ikaa*: 10; *P*. aff. *gymnodontus*: 15; *P. bifasciatus*: 30, Table S7). Muscle samples were first washed with distilled water, then lyophilized and homogenized with a mortar and pestle. We then stored all samples in 2 mL Eppendorf tubes until they were weighed into tin capsules, with each capsule containing approximately 1.6 ± 0.2 mg of dried animal tissue. Similarly, we washed all basal resources and prey items with distilled water. We then lyophilized and homogenized aquatic and terrestrial invertebrates (separated by taxonomic groups), and basal resources (Table S7). The small aquatic insects, such as Ephemeroptera, Chironomidae, Coleoptera, and Hymnoptera, underwent complete maceration. Larger prey items, including shrimps and crabs (*Aegla* sp.) were freeze‐dried and their muscle tissue was macerated. We preserved all specimens in 2 mL Eppendorf tubes before measuring and transferring them into tin capsules (1.6 ± 0.2 mg of dry animal tissue and 3.6 ± 4.2 mg of basal resources). We carried out the analysis of nitrogen (^15^N/^14^N) and carbon (^13^C/^12^C) stable isotope ratios at the Center for Nuclear Energy in Agriculture (CENA) at the University of São Paulo, Brazil. To determine the stable isotope ratios, CENA used a mass spectrometer system operating in continuous‐flow (CF‐IRMS) mode. This system was fit with a Carlo Erba elemental analyzer (CHN 1110) connected to a Delta Plus mass spectrometer (Thermo Scientific). The results of the stable isotope analysis were presented in the delta notation, which represents the deviation of stable isotope ratios (^13^C:^12^C and ^15^N:^14^N) from universal standards. Specifically, the carbon ratios were compared to the PDB limestone standard, while the nitrogen ratios were compared to atmospheric nitrogen. For the analysis of fish muscle δ^13^C, we did not correct lipids due to the low C:N ratios observed (below 3.5), indicating negligible lipid content in the samples (Hoffman et al., 2015).

### 2.4 Gross anatomy

To investigate the gross morphology of the digestive tract of tetra fishes, we measured the intestinal length with digital calipers (accuracy of 0.01 mm) and counted the number of pyloric caeca of a total of 55 adult fish specimens (*B. ikaa*: 10; *P*. aff. *gymnodontus*: 15; *P. bifasciatus*: 30). We computed the intestinal coefficient (IC) using the Hynes (1950) model, following the formula IC = IL/SL, where IL denotes the length of the intestine in mm, while SL denotes the standard length, also measured in mm. We photographed anatomical and macroscopic characteristics of the digestive tract of species using a Multipurpose Zoom Microscope Nikon AZ100M.

### 2.5 Histology and histochemistry

**FIGURE 1:**
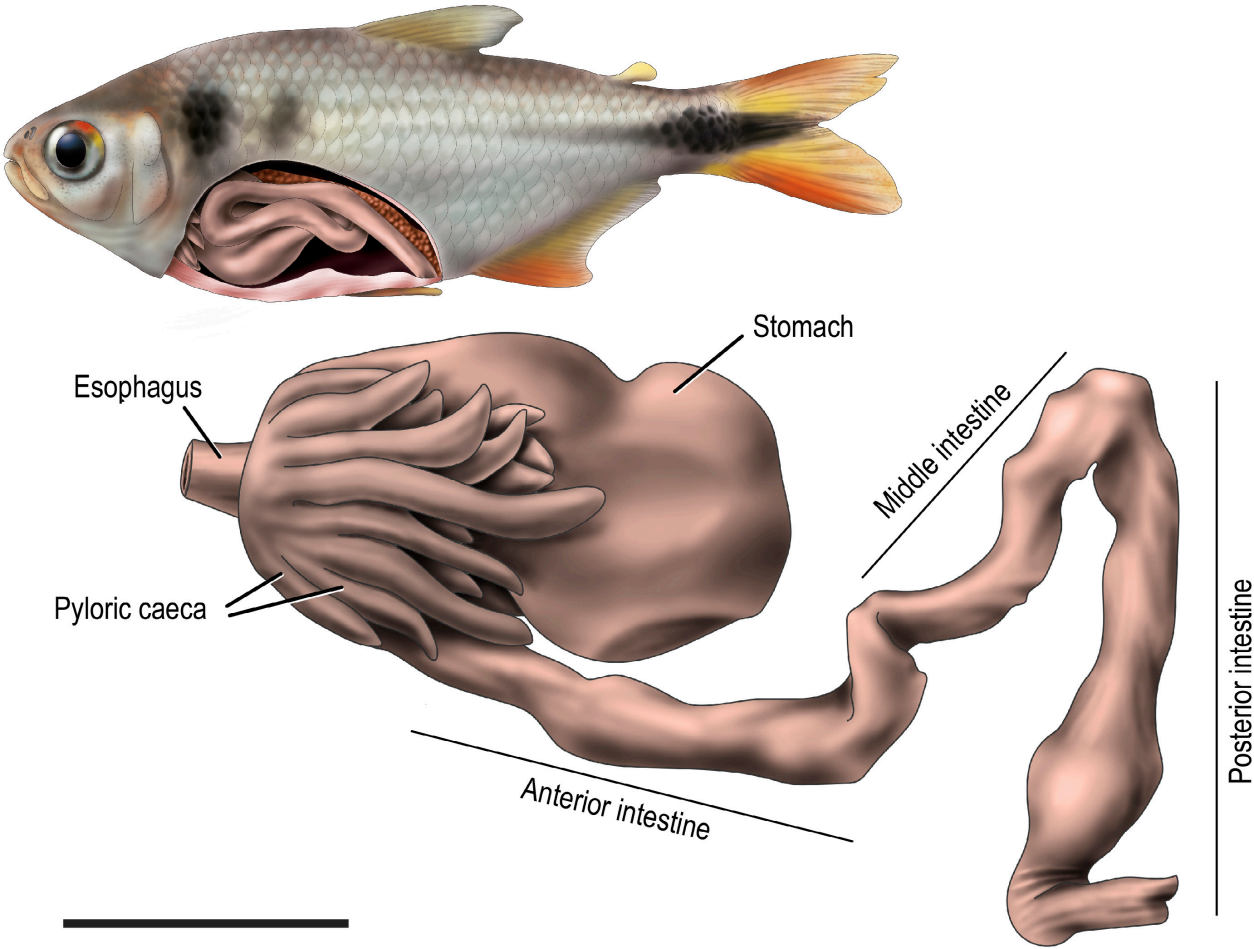
A schematic drawing of the general structure of the digestive tract of tetra fishes analyzed in this study, indicating the regions used in histological analysis. The drawing is based on a specimen of *Psalidodon bifasciatus*. Black scale bar: 1 cm. Illustrator: Presti, P.

To describe the microscopic anatomy and histology of the digestive tract of tetra fishes, we examined a total of 40 specimens (*B. ikaa*: 10; *P*. aff. *gymnodontus*: 10; *P. bifasciatus*: 20). We used the ALFAC‐fixed material and selected tissue fragments from the esophagus, stomach, and three portions of the intestine (anterior, middle, and posterior; Figure 1) of species and washed with 70% ethanol to remove the food items. These tissue fragments were then dehydrated in graded ethanol solutions and embedded in historesin (Leica®). We then made transverse and longitudinal histological sections (2–3 μm) with a Leica microtome (model RM 2145). Next, we stained the histological sections with 1% toluidine blue (TB), periodic acid Schiff (PAS) with alcian blue (AB ‐pH 2.5), and Masson's trichrome staining (MT). We used PAS + AB to detect acid and neutral mucins (Cao & Wang, 2009). Lastly, we used Masson's trichrome staining to better visualize the collagen fibers. We observed all sections using a BX60 Olympus microscope and recorded the images using an Olympus DP71 digital camera and DP Controller 3.2.1.276 software.

For a quantitative examination of the digestive tract traits, we took three histological photographs of each segment from five individuals per species. Following the methodology outlined by Bellinate et al. (2023) and Curvo et al. (2020), we used histological photographs and Fiji‐ImageJ software (Schindelin et al., 2012) to determine the number of goblet cells, thickness of the muscle layer, and the perimeter and area of the lumen, as a proxy for the absorption surface. We analyzed these variables only for the esophagus and intestine segments, as the degree of stomach fullness (i.e., full vs. empty) could cause measurement bias. Because goblet cells completely made up the esophageal epithelium, we focused on accounting for this cell type in the anterior, middle, and final segments of the intestine. We counted the number of goblet cells by randomly choosing four microvilli in each segment of the intestine. We standardized the measurements in micrometers using the scales of the histological photographs.

### 2.6 Statistical analyses

To assess any differences in the consumption of animal‐ and plant‐based food items among different species, we categorized the food items into three distinct food groups: (i) animal origin, including both aquatic and terrestrial invertebrates; (ii) plant origin, encompassing seeds and plant remnants; and (iii) undetermined origin, which consisted of detritus at various stages of decomposition and mineral particles. Subsequently, we performed a one‐way PERMANOVA analysis on an individual fish food item matrix, employing the Bray–Curtis index with 9999 permutations, as outlined by Anderson (2001).

Tissue stable isotopes can be used as tracers to determine food assimilation by different species (Nielsen et al., 2018). Nitrogen stable isotope ratios (δ^15^N) allow to estimate the trophic positions of organisms, whereas carbon stable isotope ratios (δ^13^C) allow to identify carbon sources of the diet items for aquatic consumers. Because δ^13^C changes very little with assimilation of food sources into consumers, it serves as an indicator of carbon sources, especially distinguishing between C3 and C4 plants or between aquatic and terrestrial food items. On the other hand, δ^15^N undergoes trophic fractionation, increasing by 2%–4‰ with each trophic level, allowing the determination of consumer's trophic position and food chain length (Nielsen et al., 2018). Thus, to investigate the relationships between consumption (stomach content analysis), stable isotopic composition (δ^15^N and δ^13^C values), and intestinal coefficients (numerical variable) of tetra fishes (categorical variable), we used generalized linear mixed effects models (GLMMs) that assume a normal distribution in the variation of food resources (animal and plant resources), δ^15^N and δ^13^C values. We considered the streams as a random effect, while ICs and species were treated as fixed effects. We fit the GLMMs using the *lme4* package in R programming language (Bates et al., 2015).

In addition, to determine the relative contributions of different diet sources assimilated by fishes in each site, we applied Bayesian stable isotope mixing models (Parnell et al., 2010) from the *MixSIAR* package (Stock & Semmens, 2016). Mixing models provide robust quantitative estimates of different diet contributions (Nielsen et al., 2018; Phillips et al., 2014). Here, we considered three isotopically distinct food categories: (i) animal food items, (ii) plant food items, and (iii) SOM. We used trophic discrimination factors (TDF) of 1.3 ± 0.3% for C, and 2.9 ± 0.32% for N (McCutchan et al., 2003). This enrichment is appropriate for muscle tissues of omnivorous fishes that consume mixtures of plant and animal diet (McCutchan et al., 2003). We also considered specific TDF values for plant food items (Bastos et al., 2017). Because these fishes also assimilate bacteria and other microbes inhabiting basal plant resources, we followed the method of Neres‐Lima et al. (2016) and doubled the mean discrimination factor and the variability estimate (SD) by the propagation of error (√ (2.SD2)), yielding the values 2.6 ± 0.42‰ for C, and 5.8 ± 0.45‰ for N. We fit the model with a Markov chain Monte Carlo sampling with the number of chains = 3, chain length = 100,000, burn‐in = 50,000, thin = 50, and model 4 (Resid*Process) error structure (Stock & Semmens, 2016). We examined the model convergence with diagnostic tests (Gelmin–Rubin, Heidelberger–Welch, and Geweke) and trace plots.

To examine potential differences whether the number of pyloric caeca differs among species, we conducted a one‐way ANOVA, followed by the Tukey‐HSD *post hoc* test. Regarding to the IC, considering possible differences in the standard length (SL) among species, we performed the analysis of covariance (ANCOVA) to assess whether the relationship between IC and species was influenced by the fish size. For these analyses, we used the *vegan* package (version 2.4‐6) developed by Oksanen et al. (2022) and the *car* package (Fox & Weisberg, 2019). Similarly, to test whether there are differences in the thickness of the muscular layer, perimeter and lumen area, and number of goblet cells among segments of the digestive tract and species, we applied a *two‐way* ANOVA, followed by the Tukey‐HSD post hoc test. Before conducting the tests, we confirmed that the data met the assumptions of normality and homoscedasticity. All statistical analyses were performed in the language environment R, version 4.2.1 (R Core Team, 2022).

## 3 RESULTS

### 3.1 Diet and isotopic composition

**FIGURE 2:**
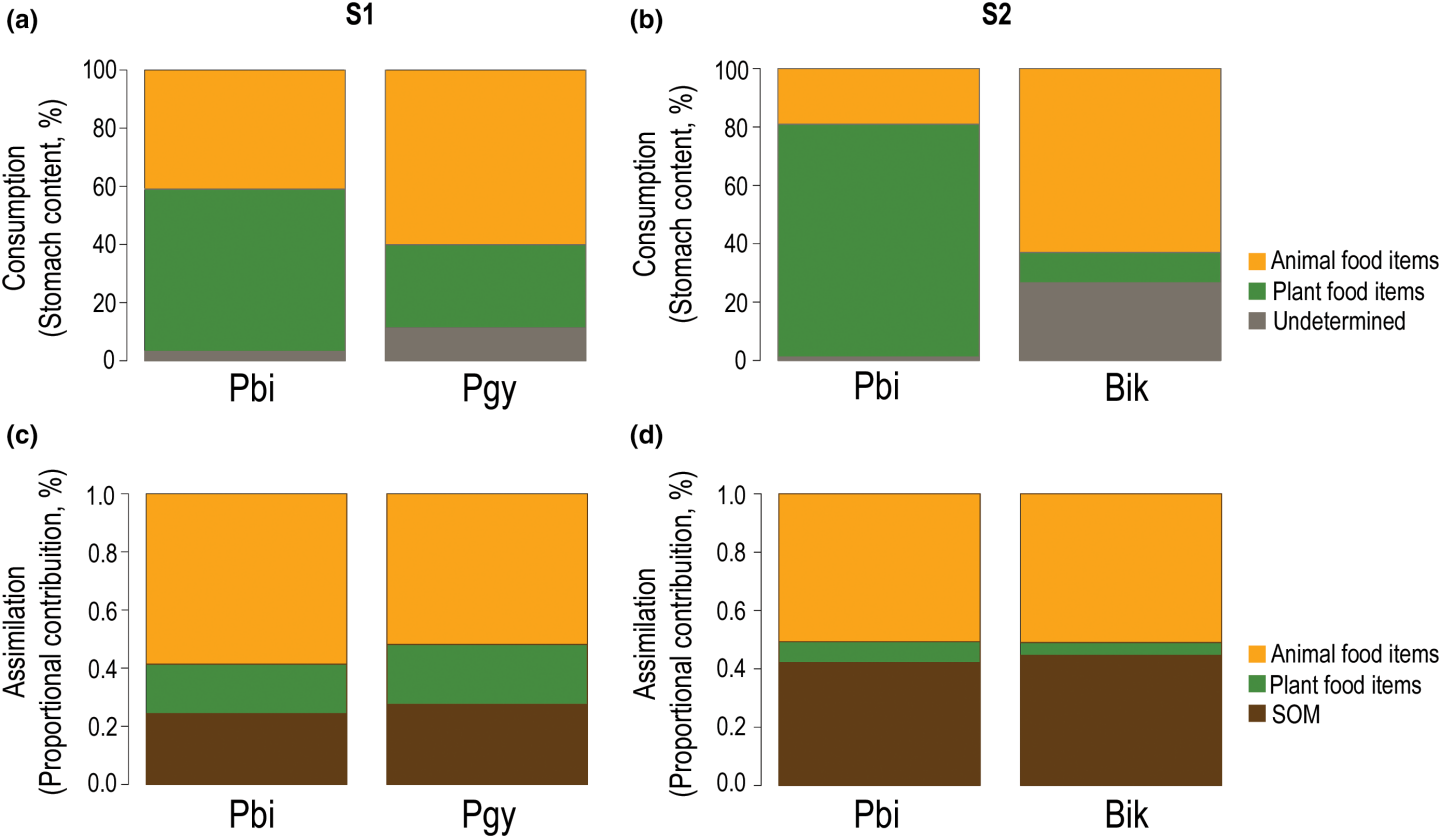
The relative contribution (%) of main food items consumed and assimilated by tetra fishes in each sampled stream (S1 and S2). (a, b) Proportions of consumed food items in diet were estimated by volume from stomach content analysis. (c, d) The assimilated diet contributions were estimated using a MixSIAR Bayesian mixing model. SOM denotes sedimentary organic matter. Species: *Bryconamericus ikaa* (Bik), *Psalidodon bifasciatus* (Pbi), and *P*. aff. *gymnodontus* (Pgy).

In agreement with our a‐priory hypothesis, there were significant differences in the food use by the three tetra fishes (Pseud‐*F* = 7.83, *p* < .001). Stomach content analysis revealed that the animal food items contributed the largest proportion to diet of *B. ikaa* (82.6%). By contrast, both *P. bifasciatus* and *P*. aff. *gymnodontus* consumed similar proportions of animal and plant food items (Figure 2). However, in contrast to stomach content analysis, there was no difference in mean δ^15^N and δ^13^C values among the three tetra species (Table 1). The relative contribution of the main food types to the muscle tissues of tetra fishes, inferred from the stable isotope mixing model, indicated that animal food items (>50%, Figure 2, Tables S7 and S8) were the main diet source assimilated by all three tetra fishes in all sites. The δ^15^N values of tetra fishes varied between 9.3 and 9.8‰ (*B. ikaa*: 9.3 ± 0.8‰; *P. bifasciatus*: 9.8 ± 1.0‰; *P*. aff. *gymnodontus*: 9.9 ± 0.51‰, mean ± standard deviation). The δ^13^C values of species varied between −24.4 and − 25.3 (*B. ikaa*: −25.3 ± 0.9‰; *P. bifasciatus*: −24.8 ± 1.4‰; *P*. aff. *gymnodontus*: 24.3 ± 0.6‰).

**TABLE 1:** The relationships between the intestinal coefficient (IC), consumption of animal and plant resources, and isotopic composition of tetra fishes obtained from generalized linear mixed effects models.

	Animal ~ IC * species + (1|stream)							δ^15^N ~ IC * species + (1 | stream)					
	Sum Sq	Mean Sq	NumDF	DenDF	*F*	Pr(>*F*)		Sum Sq	Mean Sq	NumDF	DenDF	*F*	Pr(>*F*)
IC	0.00	0.00	1	48.994	0.01	.917	IC	0.46	0.46	1	49	0.62	.437
Species	0.01	0.01	2	48.295	3.49	**.039**	Species	0.90	0.45	2	49	0.60	.551
IC:Species	0.01	0.01	2	48.578	3.57	**.036**	IC:Species	1.25	0.63	2	49	0.84	.438

	Plant ~ IC * Species + (1|Stream)							δ^13^C ~ IC * Species + (1 | Stream)					
	Sum Sq	Mean Sq	NumDF	DenDF	*F*	Pr(>*F*)		Sum Sq	Mean Sq	NumDF	DenDF	*F*	Pr(>*F*)
IC	0.00	0.00	1	49	0.2	.667	IC	0.06	0.06	1	48	0.1	.793
Species	0.00	0.00	3	48	0.2	.890	Species	3.68	1.84	2	48	2.2	.128
IC:Species	0.00	0.00	2	49	0.3	.780	IC:Species	3.35	1.68	2	48	2.0	.152

*Note*: The significant (*p* < .05) effects are given in bold.

### 3.2 Gross anatomy

**FIGURE 3:**
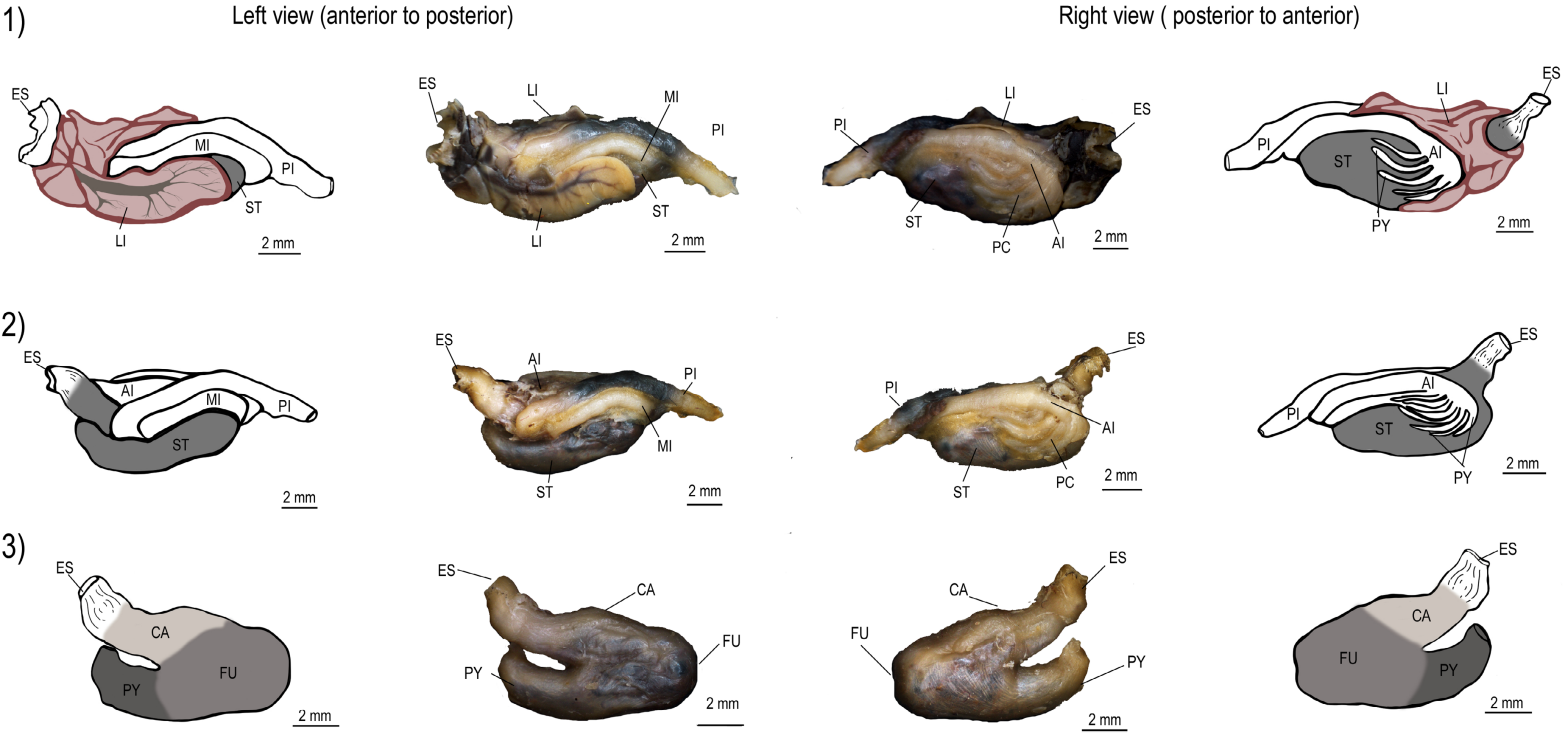
Alimentary tract of *Bryconamericus ikaa*. Digestive tracts show the liver lobes position (1), followed by dissected digestive tract showing stomach position, pyloric caeca, and intestine loops (2), and stomach shape (3). Codes: ES—esophagus; LI—liver; ST—stomach; CA—cardiac region; FU—fundic region; PY—pyloric region; PC—pyloric caeca; AI—anterior intestine; MI—middle intestine; PI—posterior intestine.

**FIGURE 4:**
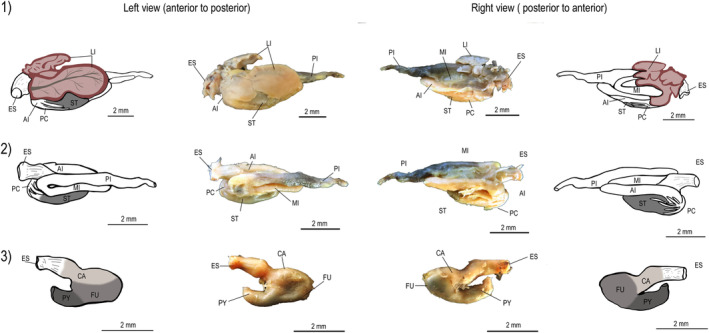
Alimentary tract of *Psalidodon* aff. *gymnodontus*. Digestive tracts show the liver lobes position (1), followed by dissected digestive tract showing stomach position, pyloric caeca, and intestine loops (2), and stomach shape (3). Codes: ES—esophagus; LI—liver; ST—stomach; CA—cardiac region; FU—fundic region; PY—pyloric region; PC—pyloric caeca; AI—anterior intestine; MI—middle intestine; PI—posterior intestine.

**FIGURE 5:**
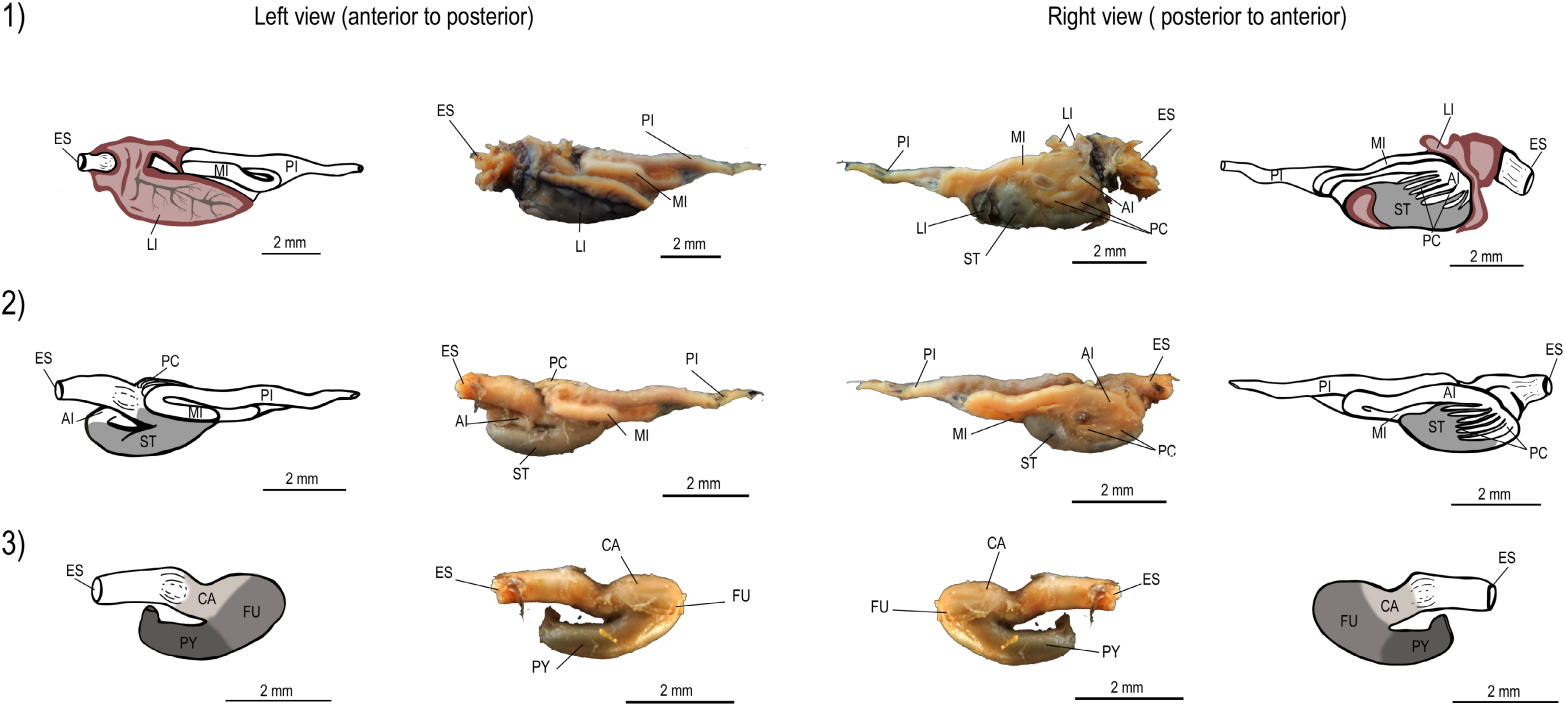
Alimentary tract of *Psalidodon bifasciatus*. Digestive tracts show the liver lobes position (1), followed by dissected digestive tract showing stomach position, pyloric caeca, and intestine loops (2), and stomach shape (3). Codes: ES—esophagus; LI—liver; ST—stomach; CA—cardiac region; FU—fundic region; PY—pyloric region; PC—pyloric caeca; AI—anterior intestine; MI—middle intestine; PI—posterior intestine.

**FIGURE 6:**
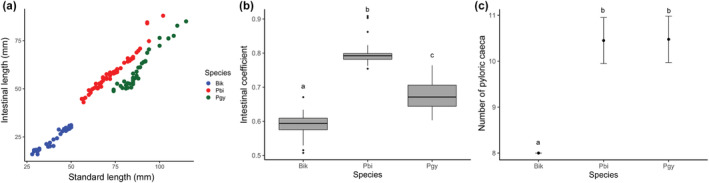
(a) Variation in standard length and macroscopic intestinal traits of tetra fishes. Species: *Bryconamericus ikaa* (Bik), *Psalidodon bifasciatus* (Pbi), and *P*. aff. *gymnodontus* (Pgy). (b) Box lower and upper endpoints represent the 25th and 75th quartiles, respectively and the horizontal bar inside each box represents median intestinal coefficient. (c) Error bars were generated based on observed number of pyloric caeca. Different letters indicate significant differences (Tukey Post‐hoc test).

Tetra fishes had a compact and cylindrical esophagus, which led to a siphonal stomach characterized by a J‐shaped saccular structure. The stomach consists of three distinct regions, namely the cardiac, fundic, and pyloric regions (Figures 3, 4, 5). Additionally, the intestinal tract consisted of four primary loops. We observed pyloric caeca in the anterior portion of the intestine, and the number of pyloric caeca differed significantly among species (*F*_2,127_ = 357.3; *p* < .0001, Figure 6). Similarly, there were significant differences in IC among species (*F*_2,124_ = 507.1; *p* < .0001). However, there was a significant interaction between species and size (SL, *F*_2,124_ = 5.10; *p* = .007), suggesting that differences in IC were associated to species size (Figure 6a). Specifically, *B. ikaa*, the smallest species, had a smaller IC and fewer pyloric caeca (8) than the other two species. In contrast, *P. bifasciatus* had a longer intestine than other species (Figure 6). Both *P. bifasciatus* and *P*. aff. *gymnodontus*, the biggest species, had a similar number of pyloric caeca (10 or 11).

### 3.3 Relationships among intestinal coefficients, consumption, and isotopic composition

As expected, there was a significant relationship between the consumption of animal food items and the IC among species (Table 1), indicating that the species with shorter intestine lengths, like *B. ikaa*, had more animals in their diet than the other two species. However, there was no significant relationship between isotopic composition and IC (Table 1), suggesting that species preferentially assimilated similar food types (i.e., animal origin) into their muscle tissues.

### 3.4 Histology and histochemistry characterization

**FIGURE 7:**
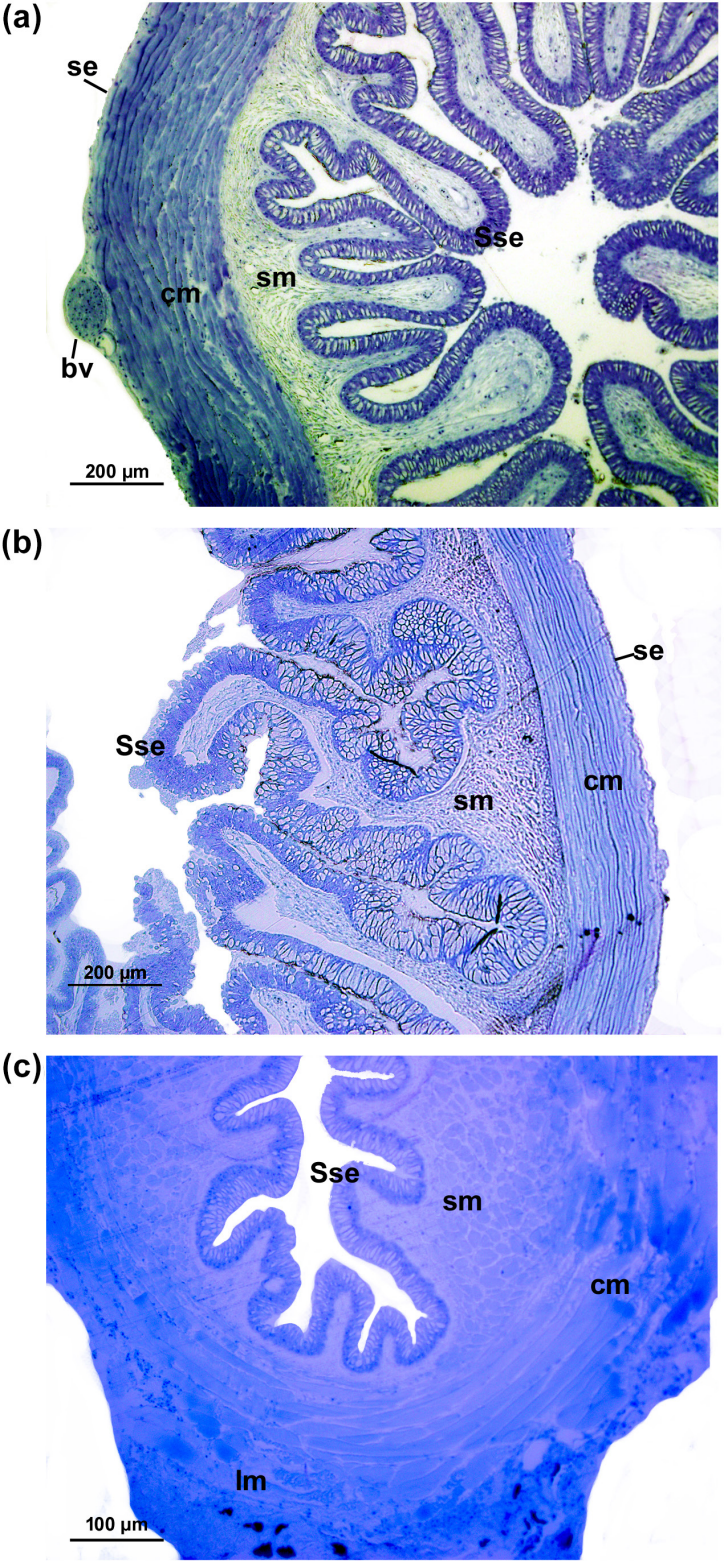
Light microscopic level showing the transversal sections of esophagus of *Psalidodon bifasciatus* (a), *P*. aff. *gymnodontus* (b) and *Bryconamericus ikaa* (c) showing the general organization: simple squamous epithelium (Sse), lamina propria (lp), circular inner of muscularis (cm), longitudinal outer of muscularis (lm), and serosa (se) with presence of blood vessel (bv). Toluidine blue (TB).

**FIGURE 8:**
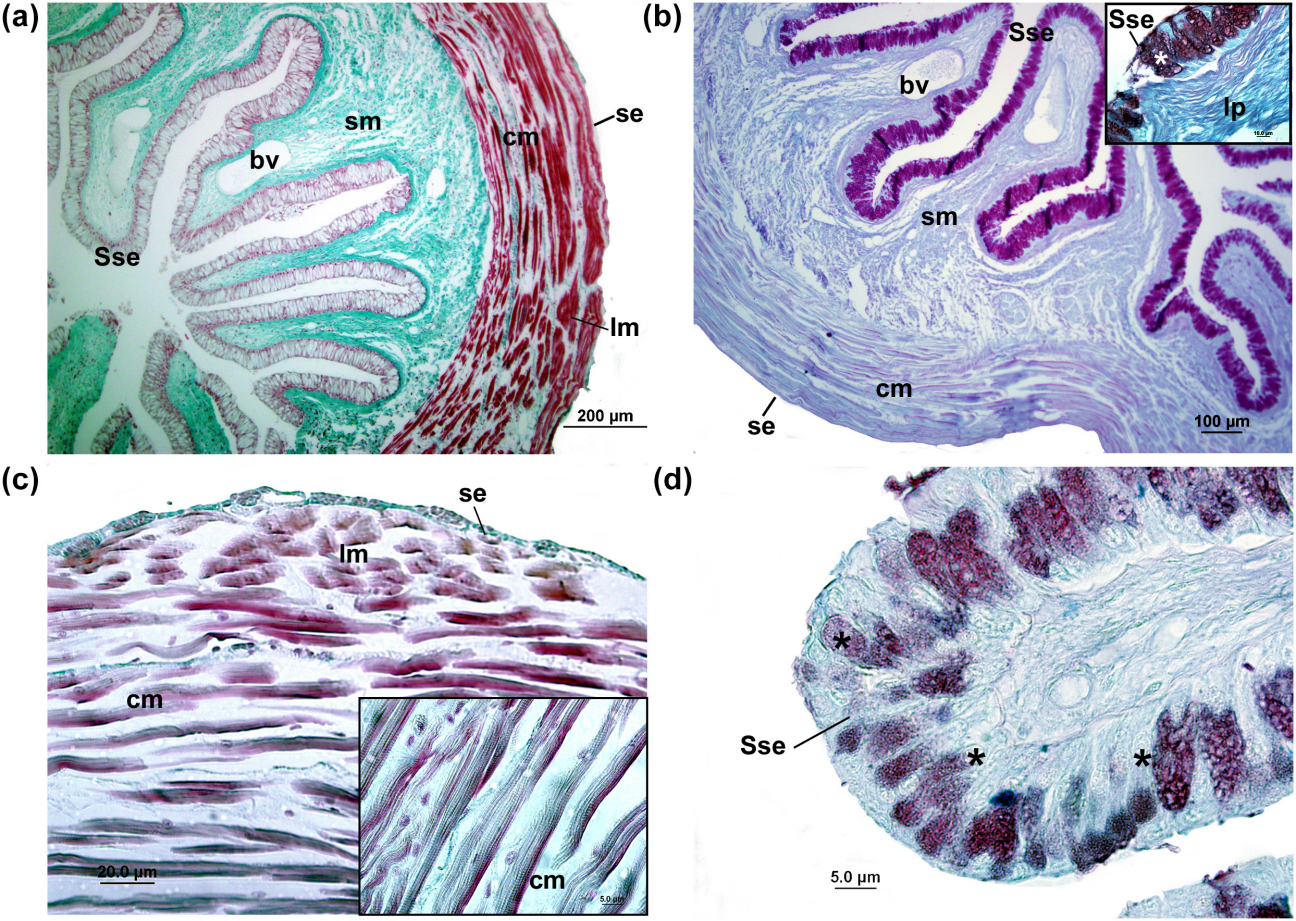
Light microscopic level showing the transversal sections of esophagus of *Psalidodon bifasciatus* using Masson's trichrome staining (a, c) and PAS + AB (b, d) reactions. In (b) and (d) amplified views of goblet cells (asterisks) in esophagus show association of acidic and neutral mucins and simple squamous epithelium. In (c) amplified view of circular inner of muscularis (mc) and longitudinal outer of muscularis (lm). Legend: simple squamous epithelium (Sse), lamina propria (lp), submucosa (sm), circular inner of muscularis (cm), longitudinal outer of muscularis (lm), and serosa (se), blood vessel (bv).

The digestive tracts of studied fishes had a similar histological composition, characterized by four distinct layers: mucosa, submucosa, muscular, and serosa. The esophageal epithelium displayed a simple squamous epithelium (Figure 7) and contained numerous mucous secreting cells (MSC) concentrated in the apical region and secreted both acid and neutral mucins (Figure 8). The submucosa layer was notably thick and comprised of dense connective tissue without any glands. This layer, greenish tones with Masson's trichrome staining (Figure 8a), had collagen fibers that provide mechanical strength. Lastly, the muscular tissue, dark red tones with Masson's trichrome staining (Figure 8a), consists of circular and longitudinal muscular layers.

**FIGURE 9:**
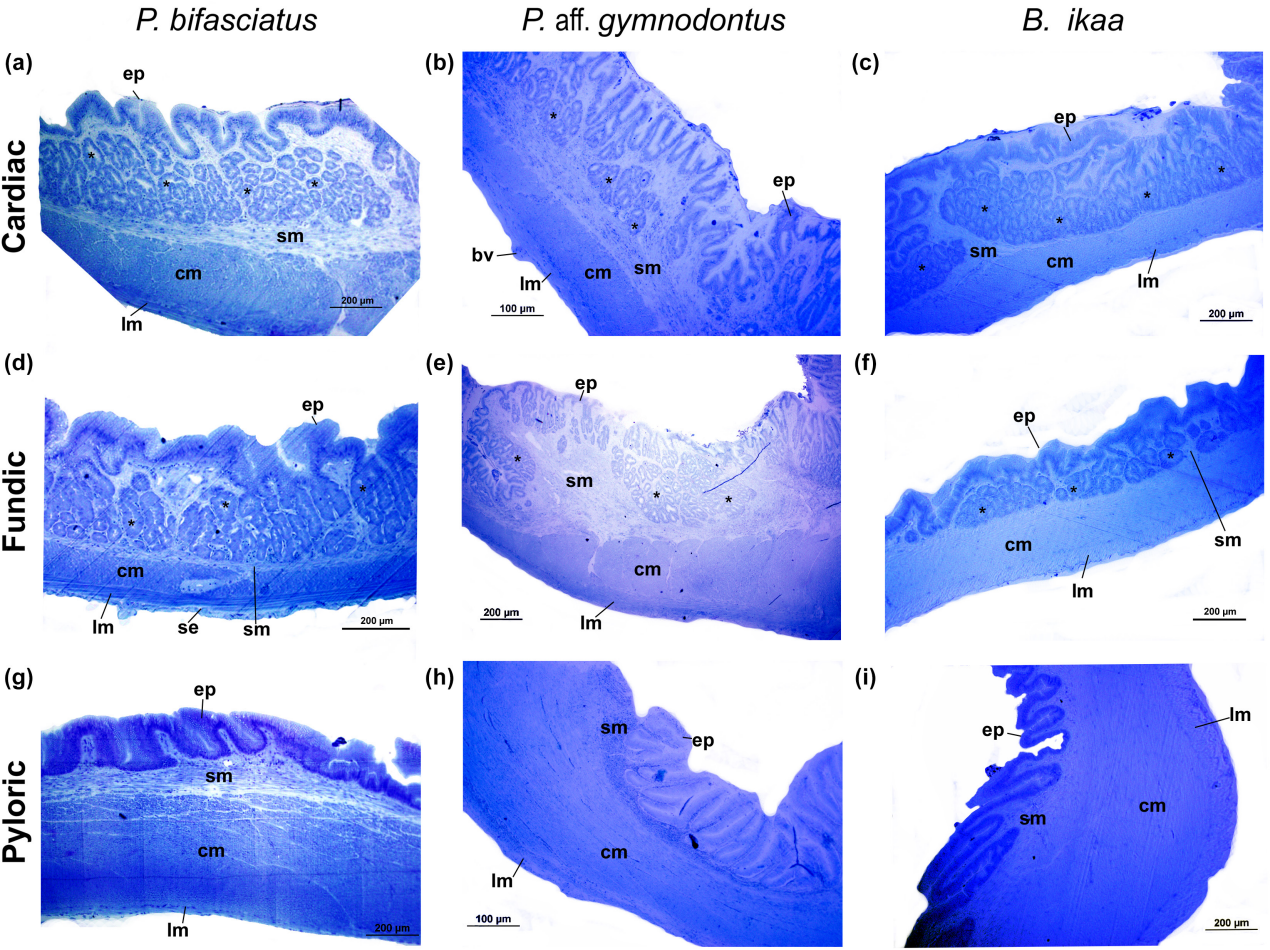
Light microscopic level showing the longitudinal sections of the cardiac (a–b–c), fundic (d–e–f) and pyloric regions of stomach of *Psalidodon bifasciatus* (a–d–g), *P*. aff. *gymnodontus* (b–e–h) and *Bryconamericus ikaa* (c–f–i) showing the general organization: columnar epithelium (ep), gastric glands (*), submucosa (sm), circular inner of muscularis (cm), longitudinal outer of muscularis (lm), serosa (se), blood vessel (bv). Toluidine blue (TB).

**FIGURE 10:**
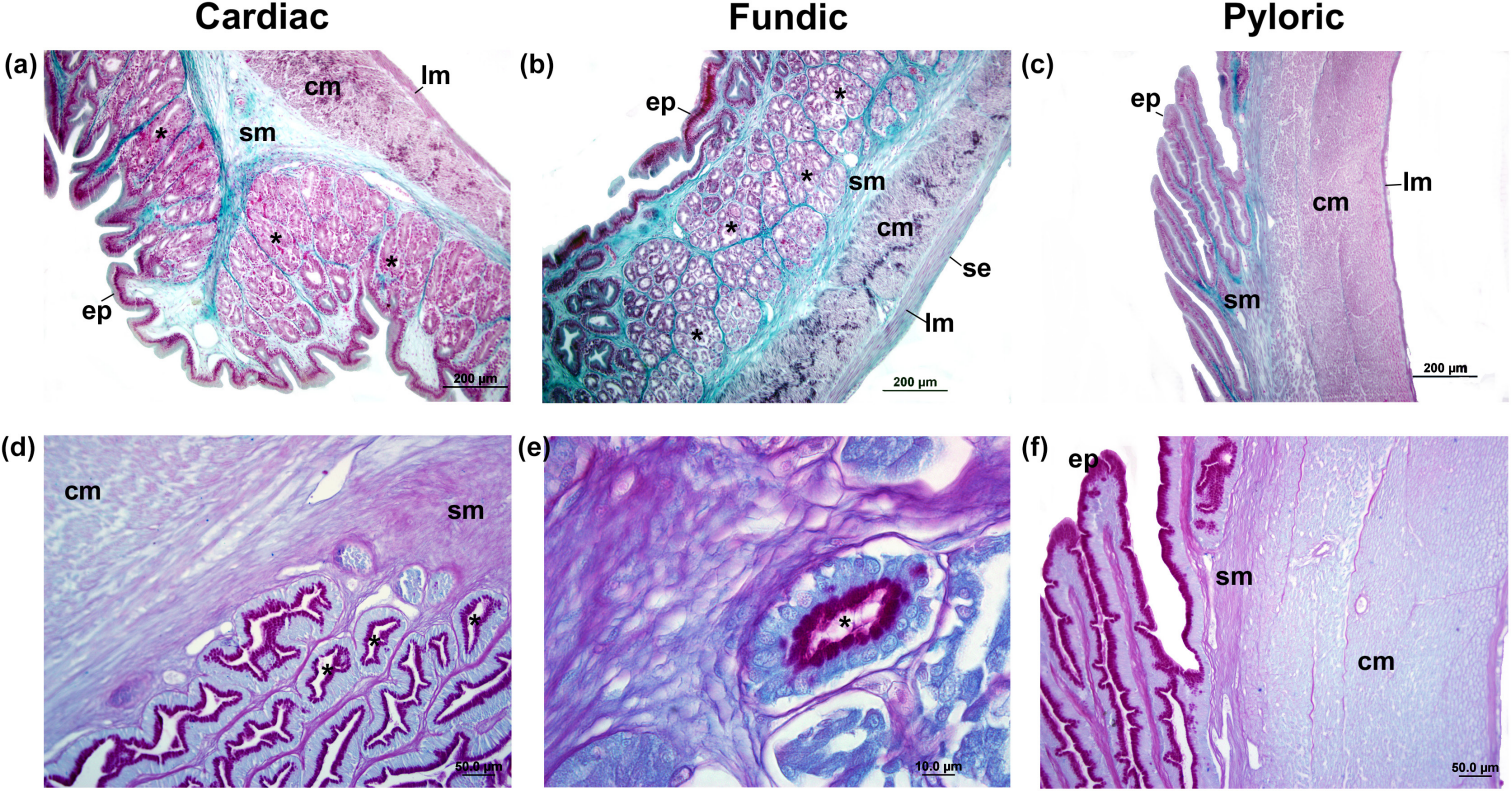
Light microscopic level showing the transversal sections of cardiac (a–d), fundic (b–e), and pyloric (c–f) regions of stomach in *Psalidodon bifasciatus* using Masson's trichrome staining (a–c) and PAS + AB (d–f) reactions. (d–e) Cardiac and fundic regions of stomach with PAS+ cells in their apical portion with gastric glands (*). The pyloric region of stomach with simple epithelium PAS+ in their apical portion, without gastric glands, and thick circular inner of muscularis. columnar epithelium (ep), submucosa (sm), circular inner of muscularis (cm), longitudinal outer of muscularis (lm), serosa (se), and blood vessel (bv).

The cardiac region of the stomach had well‐defined long folds in all three species (Figure 9). The mucosa comprised a single layer of gastric epithelium with columnar epithelial cells (Figure 9a–c), with apical mucosubstances PAS+ (Figure 10). The submucosa contained many gastric glands (Figures 9 and 10), followed by an inner circular and an outer longitudinal muscular. The fundic region had a similar structure, but the gastric gland layer gradually reduced (Figure 8d–f). Histological observations highlighted structural differences between the pyloric, cardiac, and fundic regions (Figure 9g–i). In the pyloric region, the primary variations observed were attributed to the lack of gastric glands and the existence of a substantial circular inner layer within the muscular layer (Figures 9g–i and 10c,f). The epithelium consisted of a single layer of columnar cells, which displayed positive staining for PAS+ in their apical region (Figure 9f).

**FIGURE 11:**
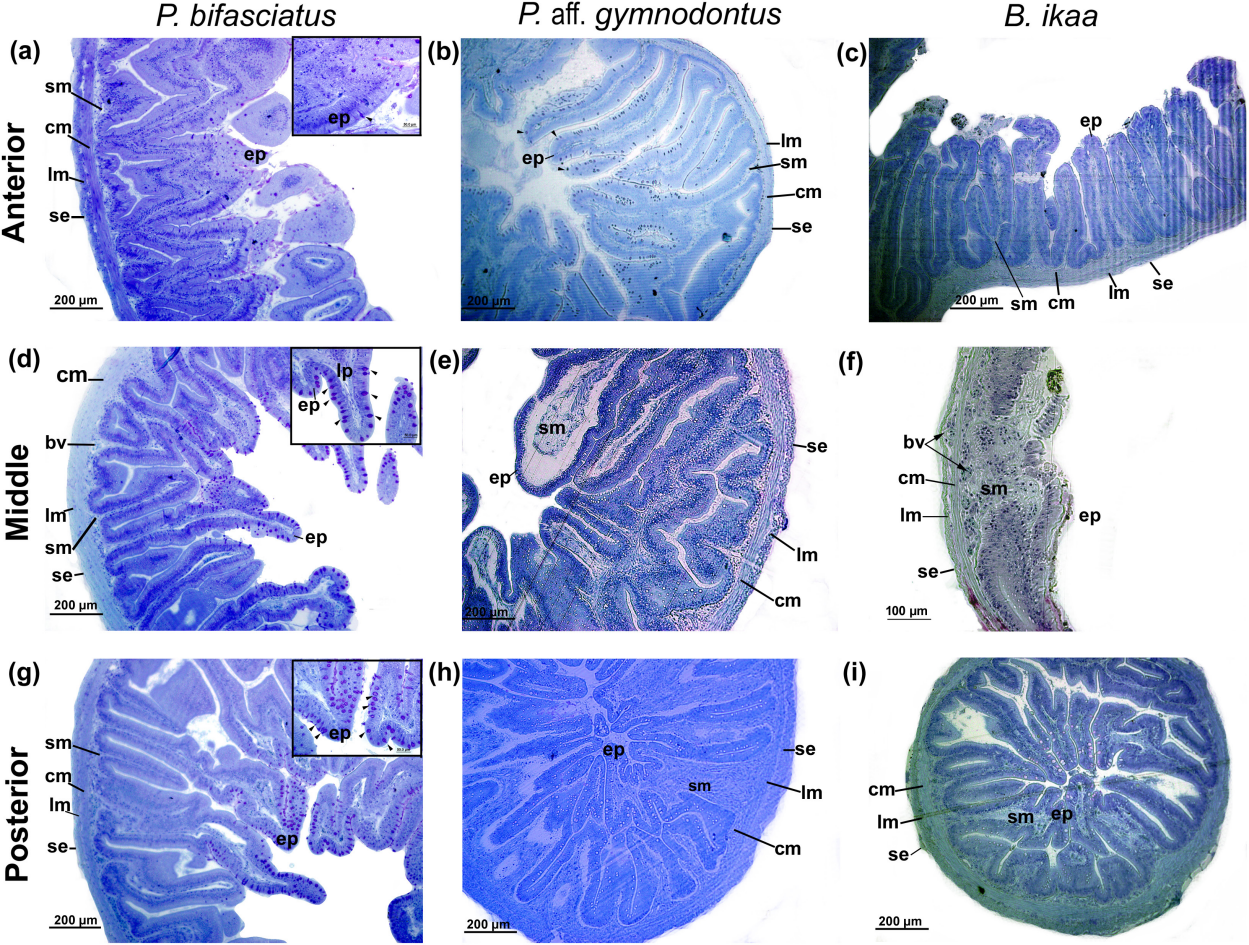
Light microscopic level showing the histological sections of the anterior (a–b–c), middle (d–e–f) and posterior intestine of *Psalidodon bifasciatus* (a–d–g), *P*. aff. *gymnodontus* (b–e–h) and *Bryconamericus ikaa* (c–f–i) showing the general organization: columnar epithelium (ep), submucosa (sm), circular inner of muscularis (cm), longitudinal outer of muscularis (lm), serosa (se), blood vessel (bv). In (b–d–g) amplified view of goblet cells (arrows head). Toluidine blue (TB).

**FIGURE 12:**
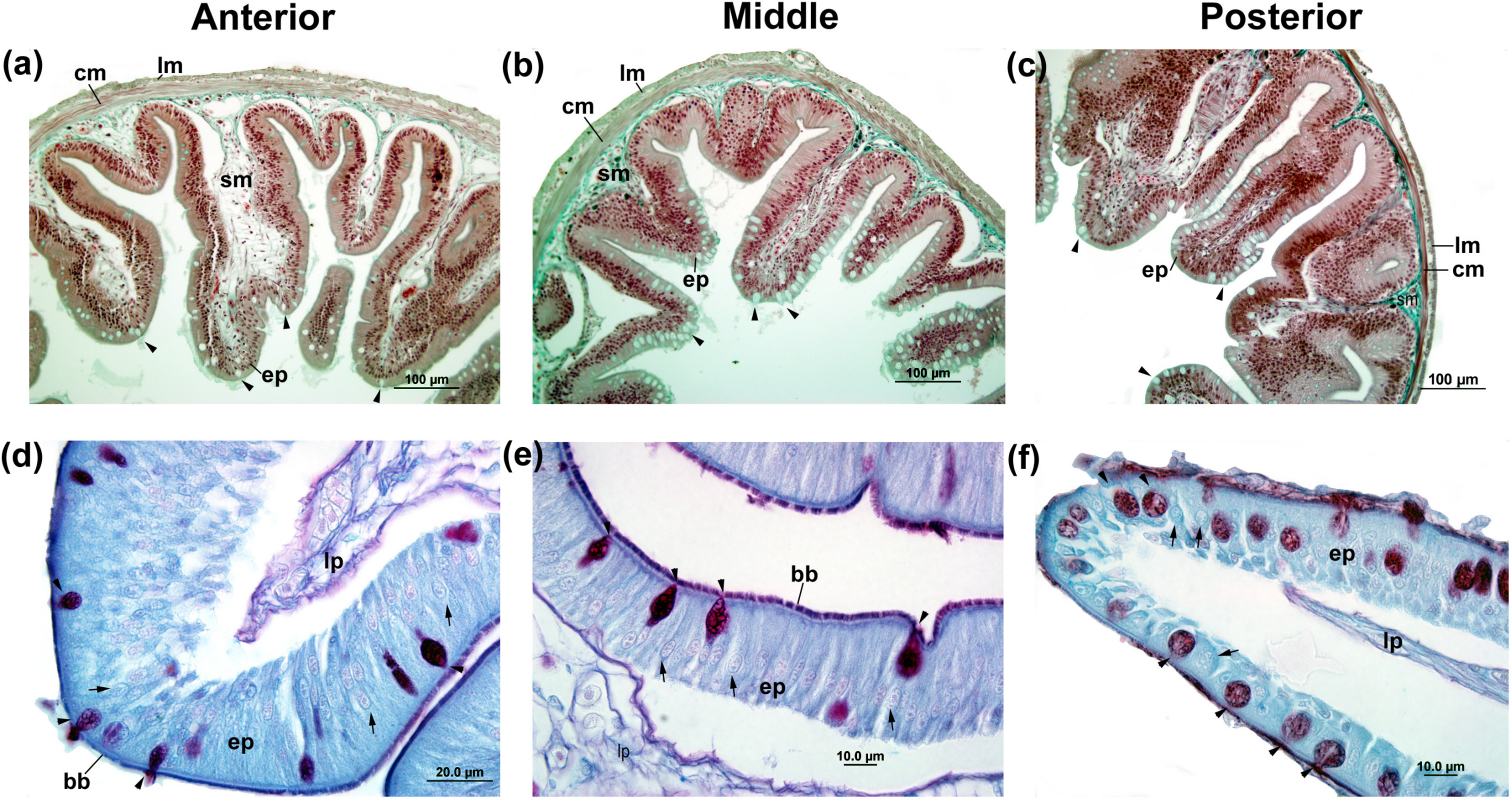
Light microscopic level showing the transversal sections of anterior (a–d), middle (b–e), and posterior (c–f) intestine of *Psalidodon bifasciatus* using Masson's trichrome staining (a–c) and PAS + AB (d–f) reactions. All portions had enterocytes (short arrow) with apical brush border (bb) and goblet cells (arrowhead). Legend: columnar epithelium (ep), lamina propria (lp), submucosa (sm), circular inner of muscularis (cm), longitudinal outer of muscularis (lm), and serosa (se).

The intestinal wall of the tetra fishes had a slender and extensively folded structure, except for the middle section in *B. ikaa* (Figure 11). The mucosa layer comprised a single layer of columnar epithelial cells known as enterocytes with a well‐developed brush border. Additionally, goblet cells were present within the mucosa layer (Figure 12d–f).

Significant differences in the histological characteristics were observed mainly among the segments of the digestive tract (Tables S1 and S2). The thickness of the muscular layer differed significantly among segments of the digestive tract (*F*_3,48_: 546.4; *p* < .0001), with a significant interaction among species and segments (Table S2). The muscular layer was significantly thicker in the esophagus, reduced in the anterior and middle segments of the intestine, and increased in the posterior intestine (Tables S1–S3). Lumen perimeter differed both among segments (*F*_3,48_: 7.51; *p* < .0001) and species (*F*_2,48_: 17.67; *p* < .0001). Specifically, the lumen perimeter tended to be greater in the final portion of the intestine and greater for *P*. aff. *gymnodontus* (Table S4). There were differences in the lumen area among segments (*F*_3,48_: 6.73; *p* = .001), with the smallest area found in the esophagus (Table S5). Regarding the number of goblet cells, there were also differences among intestinal segments (*F*_2,36_: 351.82; *p* < .0001), but neither among species (*F*_2,36_: 0.20; *p* = .82) nor in the interaction between segment and species (*F*_4,36_: 0.20; *p* = .93). The concentration of goblet cells was higher in the posterior intestine across all three species (*F*_2,36_: 351.8; *p* < .0001; Figure 12d–f, Tables S1–S6).

## 4 DISCUSSION

The studied tetra fish species differ in their proportions of animal–plant consumption yet share similar food assimilation patterns identified through stable isotope analyses. Despite differences in their digestive tract anatomy, these species maintain comparable histological structures. The intestine segments show variations in thickness, absorption surface, and goblet cell abundance. For instance, the *B. ikaa*, which is smaller in size and has shorter intestines, primarily feeds on animal‐based diets, and has fewer pyloric caeca. In contrast, larger *Psalidodon* species with longer intestines consume similar proportions of animal and plant foods and have more pyloric caeca. This suggests that despite belonging to the same family (Characidae), the evolutionary trajectories of the two genera have resulted in morphological and ecological differences that facilitated their dynamism of natural coexistence. These traits are closely related to the dietary plasticity and ecological success of each species within its environment. Therefore, the anatomy and functions of the digestive tract in these species are shaped by evolutionary, environmental, and ecological factors (Karasov et al., 2011).

Morphological traits are pivotal in understanding the trophic ecology of fish, with long intestinal length and pyloric caeca associated with herbivory, while short intestines suggest carnivorous feeding (Gerking, 1994; Karasov et al., 2011). This pattern holds for the studied species and other tetra fishes (Rincón et al., 2023). Pyloric caeca, absent in herbivores, are present in carnivores and omnivores, enhancing digestion and absorption surface area (Buddington & Diamond, 1987; Hossain & Dutta, 1996; Wilson & Castro, 2010). In our study, *B. ikaa* had the lowest number of pyloric caeca and an aquatic insectivorous feeding habit. *Psalidodon* species, characterized as omnivorous, had longer intestines and numerous pyloric caeca, consuming insects, plants, and seeds. However, despite the morphological and dietary differences between these two genera, the main assimilated resource was of animal origin. Understanding why certain fish species consume plants without utilizing them for tissue building continues to be an exciting venue for future research, promising to advance our understanding of trophic plasticity in widely distributed omnivores.

The disparity between food consumption and assimilation in tetra fishes may be governed by several mechanisms, including (i) variations in digestibility and nutritional quality (Bowen et al., 1995), (ii) the lack of specialized adaptations in their digestive tracts for breaking down and assimilating plant material (Pelster et al., 2015), (iii) the ingested plant material may contain biofilm, a primary resource assimilated into fish tissues, and (iv) ecological and behavioral factors, such as opportunistic feeding strategies and accidental consumption (Bastos et al., 2017; Bonato et al., 2018). For *P. bifasciatus* and *P*. aff. *gymnodontus*, despite the consistent intake of plant material across seasons, the rates of assimilation remained low (Neves et al., 2021). While more comparative studies are necessary to establish the incongruence between plant intake and assimilation by fish, a plausible hypothesis, alongside the gastrointestinal microbiome (Karasov et al., 2011), suggests that all animals, irrespective of their diet, require protein (Gerking, 1994). Consequently, selection should not strongly favor animals with very low protein‐processing capability (McWilliams, 2011).

Regarding the histological traits, the studied fishes had a general vertebrate pattern with the digestive tract wall (i.e., comprising four layers: mucosa, submucosa, muscular, and serosa). The esophagus and intestine shared similar histological features, featuring a mucosa with columnar epithelium and goblet cells. Notably, the muscular and submucosa layer showed a gradual thinning from the esophagus to the intestine. This submucosa layer, characterized by collagen fibers, serves a crucial function, imparting mechanical strength in the initial stages of the digestive tract, particularly in processes like swallowing (Moraes & Almeida, 2020).

The esophagus was characterized by a short and thick circular muscular layer, suggesting a specialized function for efficient food swallowing (Karasov et al., 2011). The presence of goblet cells in the esophageal epithelium potentially acts as a protective mechanism against damage caused by ingested food, since salivary glands are absent in fish (Faccioli et al., 2014; Scocco et al., 1998). Our analysis revealed the presence of acidic and neutral mucins in the esophagus, like other carnivorous and omnivorous fish species (Alonso et al., 2015; Faccioli et al., 2014; Germano et al., 2014). These mucins contribute to the thickness and stickiness of secretions, enhancing lubrication on epithelial surfaces to prevent mechanical damage and protect against potential pathogens (Abaurrea‐Equisoaín & Ostos‐Garrido, 1996; Fletcher & Grant, 1969; Humbert et al., 1984), beside immunological defense (Matheus et al., 2021). The presence of neutral mucins also indicates a possible pre‐gastric digestion process through food emulsification (Cao & Wang, 2009; Murray et al., 1996).

Tetra fishes had a distinctive J‐shaped, expandable saccular organ in the stomach, characterized by a single layer of columnar epithelium in the gastric mucosa. The J‐shaped stomach is typical of carnivorous and omnivorous fish and enables efficient digestion of large prey, while the U‐shaped stomach of herbivorous fish allows for prolonged processing of plant matter, reflecting their respective feeding behaviors (Gerking, 1994). Observed abundant neutral mucins at the apical surface of the epithelial cells are serving a protective role against autodigestion processes induced by acidic secretions from gastric glands (Bellinate et al., 2023; Cao & Wang, 2009; Faccioli et al., 2014). Gastric glands were present in the cardiac and fundic regions, playing a crucial role in prey digestion (Albrecht et al., 2001; Cao & Wang, 2009; Faccioli et al., 2014). The pyloric region exhibited unique features, including a thick smooth muscle layer, columnar cells with positive PAS staining in their apical portion, and the absence of gastric glands. The well‐developed circular smooth muscle layer in the pyloric region facilitates the mechanical force necessary to propel digested food into the intestine. In some fishes, such as mullet and trout, extreme thickening of the pyloric region resembles a gizzard, reminiscent of the avian organ used for grinding or triturating food (Wilson & Castro, 2010).

The primary function of the intestine is to finalize digestive processes initiated in the mouth and facilitate nutrient absorption (Moraes & Almeida, 2020; Wilson & Castro, 2010). Herein, the tetra fishes exhibited intestines with four main loops and pyloric caeca located in the anterior section after the pyloric stomach. Like observations in salmon species (Løkka et al., 2013) and cardinal tetra (Rincón et al., 2023), the tetra fishes have deeply folded intestines. In addition, as reported by Cho et al. (2023) for *Pseudopleuronectes yokohamae*, the thickest muscular layer and greatest perimeter and area of the lumen were observed in the posterior segment of the intestine. The intestinal lining consists of a single layer of columnar epithelium with enterocytes possessing a well‐developed brush border and goblet cells. The brush border's reactivity to PAS staining, along with the presence of glycocalyx containing neutral glycoconjugates, suggests a role in emulsifying food into chyme and aiding nutrient absorption (Bakke et al., 2010; Matheus et al., 2021; Murray et al., 1996). The alkaline phosphatase presence further supports these functions.

Goblet cells were more abundant in the posterior segment of the intestine in all three tetra fishes. The mucous secretions from these cells are important in protecting the intestinal epithelium and facilitating the smooth passage of food (Moraes & Almeida, 2020). While the specific distribution of goblet cells between the anterior and posterior sections remains unclear, their presence in both areas suggests a dual function in lubrication, aiding the movement of fecal contents and providing protection against chemical substances in the intestinal lumen, including antigens, toxins, and digestive enzymes (Wilson & Castro, 2010). This distribution pattern aligns with observations in various species across different genera, where a higher abundance of goblet cells in the posterior region is often associated with the process of defecation (Cho et al., 2023; Faccioli et al., 2014; Germano et al., 2014; Matheus et al., 2021; Rincón et al., 2023). However, exceptions exist, such as in *Leporinus* species and farmed species, where a greater abundance of goblet cells was noted in the anterior intestine, potentially linked to neutralizing stomach acids (Albrecht et al., 2001; Bellinate et al., 2023). In contrast, *Salmo salar* showed no significant differences in goblet cell abundance between intestinal segments (Løkka et al., 2013).

Herein, this study provides a detailed overview of the digestive systems of native tetra fishes and their feeding habits. Despite being omnivores, three tetra species exhibited differences in anatomy and diet. *Bryconamericus ikaa*, with a smaller body size and shorter intestines, consumed more animal food items and had fewer pyloric caeca. By contrast, *Psalidodon* species had more pyloric caeca and a balanced diet of animal and plant materials. However, all species primarily assimilated animal food into their tissues. Histologically, their digestive tracts followed a standard vertebrate plan, with variations observed in muscular layer thickness, lumen perimeter, and goblet cell numbers among different segments. These findings provide foundational insights into the digestive systems of these species, facilitating further exploration of fish feeding strategies. Like this, the study underscores the importance of ecological and morphological data in understanding the phenotypic plasticity of omnivorous consumers with implications for species coexistence.

## AUTHOR CONTRIBUTIONS

**Mayara Pereira Neves:** Conceptualization (equal); data curation (lead); formal analysis (lead); investigation (equal); methodology (equal); project administration (equal); writing – original draft (lead). **João Paulo de Arruda Amorim:** Methodology (equal); visualization (equal); writing – review and editing (equal). **Rosilene Luciana Delariva:** Conceptualization (equal); investigation (equal); methodology (equal); resources (equal); supervision (equal); validation (equal); visualization (equal); writing – review and editing (equal). **Pavel Kratina:** Resources (equal); supervision (equal); validation (equal); visualization (equal); writing – review and editing (equal). **Clarice Bernhardt Fialho:** Conceptualization (equal); funding acquisition (equal); project administration (equal); resources (equal); supervision (equal); validation (equal); visualization (equal); writing – review and editing (equal).

## CONFLICT OF INTEREST STATEMENT

The authors have no competing interests.

## Supporting information


Table S1

## Data Availability

The dataset and R‐script were archived in the DRYAD repository. Reviewers can access the files using this link: https://datadryad.org/stash/share/U0SQ2FYR4q_js9X3‐awt1NrzCgNMpMFYKdrDWtNJyFg.

## References

[ece311375-bib-0001] Abaurrea‐Equisoaín, M. A. , & Ostos‐Garrido, M. V. (1996). Cell types in the esophageal epithelium of *Anguilla anguilla* (Pisces, Teleostei). Cytochemical and ultrastructural characteristics. Micron, 27, 419–429. 10.1016/S0968-4328(96)00041-8

[ece311375-bib-0002] Albert, J. S. , Tagliacollo, V. A. , & Dagosta, F. (2020). Diversification of neotropical freshwater fishes. Annual Review of Ecology, Evolution, and Systematics, 51, 27–53. 10.1146/annurev-ecolsys-011620-031032

[ece311375-bib-0003] Albrecht, M. P. , Ferreira, M. F. N. , & Caramaschi, E. P. (2001). Anatomical features and histology of the digestive tract of two related neotropical omnivorous fishes (Characiformes; Anostomidae). Journal of Fish Biology, 58(2), 419–430. 10.1111/j.1095-8649.2001.tb02261.x

[ece311375-bib-0004] Alonso, F. , Mirande, J. M. , & Pandolfi, M. (2015). Gross anatomy and histology of the alimentary system of Characidae (Teleostei: Ostariophysi: Characiformes) and potential phylogenetic information. Neotropical Ichthyology, 13(2), 273–286. 10.1590/1982-0224-20140137

[ece311375-bib-0005] American Veterinary Medical Association . (2001). Report of the AVMA panel on euthanasia. Journal of the American Veterinary Medical Association, 218, 669–696. 11280396 10.2460/javma.2001.218.669

[ece311375-bib-0006] Anderson, M. J. (2001). A new method for non‐parametric multivariate analysis of variance. Austral Ecology, 26, 32–46. 10.1111/j.1442-9993.2001.01070.pp.x

[ece311375-bib-0007] Bakke, A. M. , Glover, C. , & Krogdahl, A. (2010). Feeding, digestion and absorption of nutrients. In M. Grosell , A. P. Farrell , & C. J. Brauner (Eds.), Fish physiology. Academic Press. 10.1016/S1546-5098(10)03002-5

[ece311375-bib-0009] Bastos, R. F. , Corrêa, F. , Winemiller, K. O. , & Garcia, A. M. (2017). Are you what you eat? Effects of trophic discrimination factors on estimates of food assimilation and trophic position with a new estimation method. Ecological Indicators, 75, 234–241. 10.1016/j.ecolind.2016.12.007

[ece311375-bib-0010] Bates, D. , Maechler, M. , Bolker, B. , & Walker, S. (2015). Fitting linear mixed‐effects models using lme4. Journal of Statistical Software, 67, 1–48. 10.48550/arXiv.1406.5823

[ece311375-bib-0068] Baumgartner, G. , Pavanelli, C. S. , Baumgartner, D. , Bifi, A. G. , Debona, T. , & Frana, V. A. (2012). Peixes do baixo rio Iguaçu. Eduem.

[ece311375-bib-0011] Bellinate, B. K. A. , Farias, K. N. N. , Do Nascimento Silva, A. L. , Siqueira, M. S. , Povh, J. A. , Franco‐Belussi, L. , & Fernandes, C. E. (2023). Intestinal morphometry and cell density features in tropical farmed fish: A methodological approach. Anatomia, Histologia, Embryologia, 52, 448–459. 10.1111/ahe.12901 36651672

[ece311375-bib-0012] Bicudo, C. E. M. , & Bicudo, R. M. T. (1970). Algas de águas continentais brasileiras chave ilustrada para identificação de gêneros (p. 228). Fundação Brasileira para o Desenvolvimento do Ensino de Ciências.

[ece311375-bib-0013] Bonato, K. O. , Burress, E. D. , Fialho, C. B. , & Armbruster, J. W. (2018). Resource partitioning among syntopic Characidae corroborated by gut content and stable isotope analyses. Hydrobiologia, 805(1), 311–324. 10.1007/s10750-017-3314-0

[ece311375-bib-0014] Bowen, S. H. , Lutz, E. V. , & Ahlgren, M. O. (1995). Dietary protein and energy as determinants of food quality: Trophic strategies compared. Ecology, 76, 899–907. 10.2307/1939355

[ece311375-bib-0015] Buddington, R. K. , & Diamond, J. (1987). Pylori ceca of fish: A “new” absorptive organ. American Journal of Physiology, 252, G65–G76. 10.1152/ajpgi.1987.252.1.G65 3812690

[ece311375-bib-0016] Cao, X. J. , & Wang, W. M. (2009). Histology and mucin histochemistry of the digestive tract of yellow catfish, *Pelteobagrus fulvidraco* . Anatomy, Histology, Embryology, 38, 254–261. 10.1111/j.1439-0264.2009.00932.x 19426437

[ece311375-bib-0017] Caputo, L. F. G. , Manso, P. A. , & Gitirana, L. B. (2011). Técnicas Histológicas. In L. Caputo , E. Molinaro , & R. Amendoeira (Eds.), Conceitos e Métodos para a Formação de Profissionais em Laboratórios de Saúde. EPSJV/Fiocruz.

[ece311375-bib-0018] Cardoso, N. N. , Firmiano, E. M. S. , Gomes, I. D. , Do Nascimento, A. A. , Sales, A. , & Araújo, F. G. (2015). Histochemical and immunohistochemical study on endocrine cells (5HT, GAS, and SST) of the gastrointestinal tract of a teleost, the characin *Astyanax bimaculatus* . Acta Histochemica, 117, 595–604. 10.1016/j.acthis.2015.05.007 26073464

[ece311375-bib-0019] Cho, J. H. , Park, J. W. , Ryu, Y. W. , Kim, K. W. , & Hur, S. W. (2023). Morphology, histology, and histochemistry of the digestive tract of the marbled flounder *Pseudopleuronectes yokohamae* . Animals, 13, 936. 10.3390/ani13050936 36899793 PMC10000053

[ece311375-bib-0020] Curvo, L. R. V. , Ferreira, M. W. , Costa, C. S. , Barbosa, G. R. C. , Uhry, S. A. , Silveira, U. S. , Andrade, G. B. , & Silva, A. R. (2020). Techniques using ImageJ for histomorphometric studies. Research, Society and Development, 9, e1459119586. 10.33448/rsd-v9i11.9586

[ece311375-bib-0021] Delariva, R. L. , & Neves, M. P. (2020). Morphological traits correlated with resource partitioning among small characin fish species coexisting in a Neotropical river. Ecology of Freshwater Fish, 29, 640–653. 10.1111/eff.12540

[ece311375-bib-0022] Dzhumaliyev, M. K. (1982). The structure of the epithelium in fishes from different taxonomic groups. Biologicheskie Nauki Kazakhstana, 1, 65–75.

[ece311375-bib-0023] Faccioli, C. K. , Chedid, R. A. , Do Amaral, A. C. , Vicentini, I. B. F. , & Vicentini, C. A. (2014). Morphology and histochemistry of the digestive tract in carnivorous freshwater *Hemisorubim platyrhynchos* (Siluriformes: Pimelodidae). Micron, 64, 10–19. 10.1016/j.micron.2014.03.011 24981208

[ece311375-bib-0024] Fanta, E. , Rios, F. S. A. , Meyer, A. A. , Grotzner, S. R. , & Zaleski, T. (2001). Chemical and visual sensory systems in feeding behavior of the Antarctic fish *Opthalmolycus amberensis* (Zoarcidae). Antarctic Record (New Zealand), 45, 27–42. 10.15094/00009151

[ece311375-bib-0025] Fletcher, T. C. , & Grant, P. T. (1969). Immunoglobulins in the serum and mucus of the plaice (*Pleuronectes platessa*). Biochemical Journal, 115, 65. 10.1042/bj1150065p 4188244 PMC1185312

[ece311375-bib-0026] Fox, J. , & Weisberg, S. (2019). An {R} companion to applied regression (3rd ed.). Sage. https://socialsciences.mcmaster.ca/jfox/Books/Companion/

[ece311375-bib-0027] Fricke, R. , Eschmeyer, W. N. , & Van der Laan, R. (2020). Eschmeyer's catalog of fishes. http://researcharchive.calacademy.org/research/ichthyology/catalog/fishcatmain.asp

[ece311375-bib-0028] Fugi, R. , & Hahn, N. S. (1991). Espectro alimentar e relações morfológicas com o aparelho digestivo de três espécies de peixes comedores de fundo do rio Paraná, Brasil. Revista Brasileira de Biologia, 51, 873–879.

[ece311375-bib-0029] Gerking, S. D. (1994). Feeding ecology of fish. Academic Press.

[ece311375-bib-0030] Germano, R. M. , Stabille, S. R. , de Britto Mari, R. , Pereira, J. N. B. , Faglioni, J. R. S. , & de Miranda Neto, M. H. (2014). Morphological characteristics of the *Pterodoras granulosus* digestive tube (Valenciennes, 1821) (Osteichthyes, Doradidae). Acta Zoologica, 95(2), 166–175. 10.1111/azo.12016

[ece311375-bib-0031] Ghilardi, M. , Schiettekatte, N. M. , Casey, J. M. , Brandl, S. J. , Degregori, S. , Mercière, A. , Morat, F. , Letourneur, Y. , Bejarano, S. , & Parravicini, V. (2021). Phylogeny, body morphology, and trophic level shape intestinal traits in coral reef fishes. Ecology and Evolution, 11(19), 13218–13231. 10.1002/ece3.8045 34646464 PMC8495780

[ece311375-bib-0032] Hellawell, J. M. , & Abel, R. (1971). A rapid volumetric method for the analysis of the food of fishes. Journal of Fish Biology, 3(1), 29–37. 10.1111/j.10958649.1971.tb05903.x

[ece311375-bib-0033] Hoffman, J. C. , Sierszen, M. E. , & Cotter, A. M. (2015). Fish tissue lipid‐C: N relationships for correcting δ^13^C values and estimating lipid content in aquatic food‐web studies. Rapid Communications in Mass Spectrometry, 29, 2069–2077. 10.1002/rcm.7367 26443408

[ece311375-bib-0034] Hossain, A. M. , & Dutta, H. M. (1996). Phylogeny, ontogeny, structure and function of digestive tract appendages (caeca) in teleost fish. In J. S. Datta Munshi & H. M. Dutta (Eds.), Fish morphology (pp. 59–76). AA Balkema Publishers.

[ece311375-bib-0035] Humbert, W. , Kirsch, R. , & Meister, M. F. (1984). Scanning electron microscopic study of the oesophageal mucous layer in the eel *Anguilla anguilla* L. Journal of Fish Biology, 25, 117–122. 10.1111/j.1095-8649.1984.tb04856.x

[ece311375-bib-0036] Hynes, H. B. N. (1950). The food of freshwater sticklebacks (*Gasteroteus aculeatus* and *Pygosteus pungitius*), with a review of methods used in studies of the food of fishes. Journal of Animal Ecology, 19, 35–58. 10.2307/1570

[ece311375-bib-0037] Hyslop, E. J. (1980). Stomach contents analysis—A review of methods and their application. Journal of Fish Biology, 17(4), 411–429. 10.1111/j.1095-8649.1980.tb02775.x

[ece311375-bib-0038] Javahery, S. , Nekoubin, H. , & Moradlu, A. H. (2012). Effect of anesthesia with clove oil in fish. Fish Physiology and Biochemistry, 38, 1545–1552. 10.1007/s10695-012-9682-5 22752268

[ece311375-bib-0039] Karasov, W. H. , Martinez del Rio, C. , & Caviedes‐Vidal, E. (2011). Ecological physiology of diet and digestive systems. Annual Review of Physiology, 73, 69–93. 10.1146/annurev-physiol-012110-142152 21314432

[ece311375-bib-0040] Løkka, G. , Austbø, L. , Falk, K. , Bjerkås, I. , & Koppang, E. O. (2013). Intestinal morphology of the wild Atlantic salmon (*Salmo salar*). Journal of Morphology, 274(8), 859–876. 10.1002/jmor.20142 23520065

[ece311375-bib-0042] Matheus, V. A. , Faccioli, C. K. , Chedid, R. A. , Senhorini, J. A. , Franceschini‐Vicentini, I. B. , & Vicentini, C. A. (2021). Morphological and histochemical features of the digestive tract of *Leiarius marmoratus* (Gill, 1870). Journal of Fish Biology, 99(5), 1622–1631. 10.1111/jfb.14868 34331469

[ece311375-bib-0043] McCutchan, J. H., Jr., Lewis, W. M., Jr., Kendall, C. , & McGrath, C. C. (2003). Variation in trophic shift for stable isotope ratios of carbon, nitrogen, and sulfur. Oikos, 102, 378–390. 10.1034/j.1600-0706.2003.12098.x

[ece311375-bib-0044] McWilliams, S. R. (2011). Ecology of vertebrate nutrition. In eLS. John Wiley & Sons Ltd. 10.1002/9780470015902.a0003211.pub2

[ece311375-bib-0045] Moraes, G. , & Almeida, L. C. (2020). Nutrition and functional aspects of digestion in fish. In B. Baldisserotto , E. C. Urbinati , & J. E. P. Cyrino (Eds.), Biology and physiology of freshwater neotropical fish. Academic Press. 10.1016/B978-0-12-815872-2.00011-7

[ece311375-bib-0046] Mugnai, R. , Nessimian, J. L. , & Baptista, D. F. (2010). Manual de identificação de macroinvertebrados aquáticos do estado do Rio de Janeiro. Technical Boocks.

[ece311375-bib-0047] Murray, H. M. , Wright, G. M. , & Goff, G. P. (1996). A comparative histological and histochemical study of the post‐gastric alimentary canal from three species of Pleuronectid, the Atlantic halibut, the yellowtail flounder and the winter flounder. Journal of Fish Biology, 48, 187–206. 10.1111/j.1095-8649.1996.tb01112.x

[ece311375-bib-0048] Neres‐Lima, V. , Brito, E. F. , Krsulović, F. A. , Detweiler, A. M. , Hershey, A. E. , & Moulton, T. P. (2016). High importance of autochthonous basal food source for the food web of a Brazilian tropical stream regardless of shading. International Review of Hydrobiology, 101, 132–142. 10.1002/iroh.201601851

[ece311375-bib-0049] Neves, M. P. , Delariva, R. L. , Perkins, D. M. , Fialho, C. B. , & Kratina, P. (2023). Trophic plasticity of omnivorous fishes in natural and human‐dominated landscapes. Limnology and Oceanography., 69, 189–202. 10.1002/lno.12467

[ece311375-bib-0050] Neves, M. P. , Kratina, P. , Delariva, R. L. , Jones, J. I. , & Fialho, C. B. (2021). Seasonal feeding plasticity can facilitate coexistence of dominant omnivores in Neotropical streams. Reviews in Fish Biology and Fisheries, 31, 417–432. 10.1007/s11160-021-09648-w

[ece311375-bib-0051] Nielsen, M. J. , Clare, L. E. , Hayden, B. , Brett, T. M. , & Kratina, P. (2018). Diet tracing in ecology: Method comparison and selection. Methods in Ecology and Evolution, 9, 278–291. 10.1111/2041-210X.12869

[ece311375-bib-0070] Oksanen, J. , Simpson, G. , Blanchet, F. , Kindt, R. , Legendre, P. , Minchin, P. , O'Hara, R. , Solymos, P. , Stevens, M. , Szoecs, E. , Wagner, H. , Barbour, M. , Bedward, M. , Bolker, B. , Borcard, D. , Carvalho, G. , Chirico, M. , De Caceres, M. , Durand, S. , … Weedon, J. (2022). Vegan: Community Ecology Package. R package version 2.6‐4. https://CRAN.R‐project.org/package=vegan

[ece311375-bib-0069] Ota, R. R. , Deprá, G. D. C. , Graça, W. J. D. , & Pavanelli, C. S. (2018). Peixes da planície de inundação do alto rio Paraná e áreas adjacentes: revised, annotated and updated. Neotropical Ichthyology, 16, e170094.

[ece311375-bib-0054] Parnell, A. C. , Inger, R. , Bearhop, S. , & Jackson, A. L. (2010). Source partitioning using stable isotopes: Coping with too much variation. PLoS One, 5, e9672. 10.1371/journal.pone.0009672 20300637 PMC2837382

[ece311375-bib-0055] Pelster, B. , Wood, C. M. , Speers‐Roesch, B. , Driedzic, W. R. , Almeida‐Val, V. , & Val, A. (2015). Gut transport characteristics in herbivorous and carnivorous serrasalmid fish from ion‐poor Rio Negro water. Journal of Comparative Physiology B, 185, 225–241. 10.1007/s00360-014-0879-z 25528145

[ece311375-bib-0056] Phillips, D. L. , Inger, R. , Bearhop, S. , Jackson, A. L. , Moore, J. W. , Parnell, A. C. , Semmens, B. X. , & Ward, E. J. (2014). Best practices for use of stable isotope mixing models in food‐web studies. Canadian Journal of Zoology, 92, 823–835. 10.1139/cjz-2014-0127

[ece311375-bib-0057] Pini, S. F. R. , Abelha, M. C. F. , Kashiwaqui, E. A. L. , Delariva, R. L. , Makrakis, S. , & Makrakis, M. (2019). Food resource partitioning among species of *Astyanax* (Characiformes: Characidae) in the low Iguaçu River and tributaries, Brazil. Neotropical Ichthyology, 17, e190028. 10.1590/1982-0224-20190028

[ece311375-bib-0058] Quirino, B. A. , Carniatto, N. , Gaiotto, J. V. , & Fugi, R. (2015). Seasonal variation in the use of food resources by small fishes inhabiting the littoral zone in a Neotropical floodplain lake. Aquatic Ecology, 49, 431–440. 10.1007/s10452-015-9535-2

[ece311375-bib-0059] R Core Team . (2022). R: A language and environment for statistical computing. R Foundation for Statistical Computing. http://www.r‐project.org/index.html

[ece311375-bib-0060] Ribble, D. O. , & Smith, M. H. (1983). Relative intestine length and feeding ecology of freshwater fishes. Growth, 47(3), 292–300. 6642249

[ece311375-bib-0061] Rincón, L. , Redondo, F. , Kobrinsky, W. , Pandolfi, M. , & Pozzi, A. G. (2023). Morphological study of the digestive tract of the cardinal tetra, *Paracheirodon axelrodi* (Characiformes: Characidae). Neotropical Ichthyology, 21, e220092. 10.1590/1982-0224-2022-0092

[ece311375-bib-0062] Schindelin, J. , Arganda‐Carreras, I. , Frise, E. , Kaynig, V. , Longair, M. , Pietzsch, T. , Preibisch, S. , Rueden, C. , Saalfeld, S. , Schmid, B. , Tinevez, J. Y. , White, D. J. , Hartenstein, V. , Eliceiri, K. , Tomancak, P. , & Cardona, A. (2012). Fiji: An open‐source platform for biological‐image analysis. Nature Methods, 9, 676–682. 10.1038/nmeth.2019 22743772 PMC3855844

[ece311375-bib-0063] Scocco, P. , Accili, D. , Menghi, G. , & Ceccarelli, P. (1998). Unusual glycoconjugates in the esophagus of a tilapine polyhybrid. Journal of Fish Biology, 53, 39–48. 10.1111/j.1095-8649.1998.tb00107.x

[ece311375-bib-0064] Stock, B. C. , & Semmens, B. X. (2016). MixSIAR GUI user manual. Version 3.1.

[ece311375-bib-0065] Vazzoler, A. E. A. M. (1996). Biologia da reprodução de peixes teleóteos: teoria e prática. Eduem.

[ece311375-bib-0066] Végaz‐Velez, M. (1972). La structure histologique typique du tube digestif dês poissons télèosteens. Tethys, 4, 163–174.

[ece311375-bib-0067] Wilson, J. M. , & Castro, L. F. C. (2010). Morphological diversity of the gastrointestinal tract in fishes. In M. Grosell , A. P. Farrell , & C. J. Brauner (Eds.), Fish physiology: The multifunctional gut of fish (Vol. 30, pp. 1–55). Academic Press. 10.1016/S1546-5098(10)03001-3

