# Supplementary material for: Linking anatomical and histological traits of the digestive tract to resource consumption and assimilation of omnivorous tetra fishes
Source: Ecol Evol. 2024 May 2;14(5):e11375. doi: 10.1002/ece3.11375 (PMC11066418; doi:10.1002/ece3.11375)
Supplement: Supplementary file 1 — Table S1 [file ECE3-14-e11375-s001.docx]

**Supplementary material**

**Linking anatomical and histological traits of the digestive tract to resource consumption and assimilation of omnivorous tetra fishes**

*Mayara Pereira Neves^1,5,*^, João Paulo de Arruda Amorim^2^, Rosilene Luciana Delariva^3^, Pavel Kratina^4^, Clarice Bernhardt Fialho^5^*

*^1^Department of Biosciences, Rice University, Houston, TX, United States*

*^2^Laboratório de Biologia Tecidual e da Reprodução, Universidade Estadual do Oeste do Paraná, Centro de Ciências Biológicas e da Saúde, Rua Universitária 2069, 85819-110. Cascavel, PR, Brazil*

*^3^Laboratório de Ictiologia, Ecologia e Biomonitoramento, Universidade Estadual do Oeste do Paraná, Centro de Ciências Biológicas e da Saúde, Rua Universitária 2069, 85819-110. Cascavel, PR, Brazil*

*^4^School of Biological and Behavioural Sciences, Queen Mary University of London, London, United Kingdom*

*^5^Programa de Pós-Graduação em Biologia Animal, Departamento de Zoologia, Instituto de Biociências, Universidade Federal do Rio Grande do Sul, Porto Alegre, RS, Brazil*

***Corresponding author**: Mayara P. **Neves** (ORCID: 0000-0003-2523-3874) - Department of Biosciences, Rice University, 6100 Main St, Houston, TX 77005, United States. Email: [mn70@rice.edu](mailto:mn70@rice.edu), [mayara-nevesbio@hotmail.com](mailto:mayara-nevesbio@hotmail.com)

**List of contents**

**Table S1** – Summary of digestive tract traits of omnivorous tetra fishes from neotropical streams, southern Brazil.

**Table S2** – Digestive tract traits differences of omnivorous tetra fishes from neotropical streams, southern Brazil, obtained from Analysis of Variance (ANOVA).

**Table S3** – *Post hoc* of Analysis of Variance (ANOVA) applied to muscular layer of digestive tract of omnivorous tetra fishes from neotropical streams, southern Brazil.

**Table S4** – *Post hoc* of Analysis of Variance (ANOVA) applied to lumen perimeter of digestive tract of omnivorous tetra fishes from neotropical streams, southern Brazil.

**Table S5** – *Post hoc* of Analysis of Variance (ANOVA) applied to lumen area of digestive tract of omnivorous tetra fishes from neotropical streams, southern Brazil.

**Table S6** – *Post hoc* of Analysis of Variance (ANOVA) applied to the number of goblet cells in different segments of the intestine of omnivorous tetra fishes from neotropical streams, southern Brazil.

**Table S7** – Total number of samples (n) of the basal resources, prey and omnivorous fish sampled for isotopic analyses.

**Table S8** – Summary of relative isotopic contribution of food resources to the diet of omnivorous tetra fishes from neotropical streams, estimated by a MixSIAR Bayesian mixing model.

**Table S1** – Summary of digestive tract traits of omnivorous tetra fishes from neotropical streams, southern Brazil. Codes: AI: anterior intestine; IM: medium intestine; IF: posterior intestine.

|  |  | Muscular layer (μm) | |  | Lumen perimeter (μm) | |  | Lumen area (μm) | |  | Number of goblet cells | |
| --- | --- | --- | --- | --- | --- | --- | --- | --- | --- | --- | --- | --- |
| Species | Segment | Mean | SD |  | Mean | SD |  | Mean | SD |  | Mean | SD |
| Bik | Esophagus | 252.17 | 39.12 |  | 10,294.07 | 1,403.36 |  | 598,856.22 | 436,935.83 |  | - | - |
|  | AI | 39.00 | 1.96 |  | 12,122.72 | 150.83 |  | 1,591,235.57 | 4,492.75 |  | 15.51 | 2.36 |
|  | IM | 25.26 | 2.99 |  | 6,574.89 | 24.52 |  | 868,270.36 | 413.58 |  | 20.16 | 2.89 |
|  | IF | 54.15 | 7.79 |  | 12,900.21 | 99.85 |  | 279,230.38 | 1,514.27 |  | 51.82 | 4.64 |
| Pbi | Esophagus | 290.59 | 36.90 |  | 11,724.30 | 2,387.75 |  | 404,500.26 | 69,295.83 |  | - | - |
|  | AI | 35.25 | 9.76 |  | 14,247.18 | 1,972.33 |  | 1,271,058.02 | 684,121.72 |  | 17.98 | 4.30 |
|  | IM | 32.48 | 8.54 |  | 9,379.42 | 1,962.61 |  | 1,097,229.12 | 554,145.27 |  | 20.16 | 2.89 |
|  | IF | 26.46 | 6.99 |  | 28,871.10 | 24,788.78 |  | 1,567,993.57 | 1,381,745.00 |  | 51.82 | 4.64 |
| Pgy | Esophagus | 200.38 | 18.66 |  | 10,556.16 | 159.16 |  | 293,851.55 | 1,192.01 |  | - | - |
|  | AI | 54.96 | 3.46 |  | 30,384.82 | 141.20 |  | 848.372.03 | 789.21 |  | 17.98 | 4.30 |
|  | IM | 48.11 | 2.53 |  | 29,397.21 | 77.87 |  | 1,046,503.55 | 1,766.93 |  | 20.16 | 2.89 |
|  | IF | 40.41 | 4.74 |  | 29,725.87 | 10,043.87 |  | 782.893.55 | 419,102.12 |  | 51.82 | 4.64 |

**Table S2** – Digestive tract traits differences of omnivorous tetra fishes from neotropical streams, southern Brazil, obtained from Analysis of Variance (ANOVA). Significant p-values are in bold.

|  | Muscular layer | | | | |
| --- | --- | --- | --- | --- | --- |
|  | Df | Sum Sq | Mean Sq | F value | Pr(>F) |
| Segment | 3 | 487,894 | 162,631 | 546.398 | **<0.0001** |
| Species | 2 | 1,079 | 540 | 1.813 | 0.174 |
| Segment:Species | 6 | 23,791 | 3,965 | 13.322 | **<0.0001** |
| Residuals | 48 | 14,287 | 298 |  |  |
|  | Lumen perimeter | | | | |
|  | Df | Sum Sq | Mean Sq | F value | Pr(>F) |
| Segment | 3 | 1,372,000,000 | 457,500,000 | 7.5110 | **<0.0001** |
| Species | 2 | 2,153,000,000 | 1,077,000,000 | 17.6750 | **<0.0001** |
| Segment:Species | 6 | 1,297,000,000 | 216,200,000 | 3.5500 | **0.005** |
| Residuals | 48 | 2,923,000,000 | 60,910,000 |  |  |
|  | Lumen area | | | | |
|  | Df | Sum Sq | Mean Sq | F value | Pr(>F) |
| Segment | 3 | 5,143,000,000,000.0 | 1,714,000,000,000.0 | 6.732 | **0.001** |
| Species | 2 | 1,256,000,000,000.0 | 628,100,000,000.0 | 2.467 | 0.096 |
| Segment:Species | 6 | 4,733,000,000,000.0 | 788,900,000,000.0 | 3.098 | 0.012 |
| Residuals | 48 | 12,220,000,000,000.0 | 254,600,000,000.0 |  |  |
|  | Number of goblet cells | | | | |
|  | Df | Sum Sq | Mean Sq | F value | Pr(>F) |
| Segment | 2 | 11,024 | 5,512 | 351.817 | **<0.0001** |
| Species | 2 | 6 | 3 | 0.204 | 0.816 |
| Segment:Species | 4 | 13 | 3 | 0.204 | 0.934 |
| Residuals | 36 | 564 | 16 |  |  |

**Table S3** – *Post hoc* of Analysis of Variance (ANOVA) applied to muscular layer of digestive tract of omnivorous tetra fishes from neotropical streams, southern Brazil. Significant p-values are in bold.

| Segment/Species | diff | lwr | upr | p adj |
| --- | --- | --- | --- | --- |
| IF:Bik-Esophagus:Bik | -198.02 | -235.49 | -160.55 | **0.000** |
| II:Bik-Esophagus:Bik | -213.17 | -250.64 | -175.70 | **0.000** |
| IM:Bik-Esophagus:Bik | -226.91 | -264.38 | -189.45 | **0.000** |
| Esophagus:Pbi-Esophagus:Bik | 38.42 | 0.96 | 75.89 | **0.040** |
| IF:Pbi-Esophagus:Bik | -225.71 | -263.17 | -188.24 | **0.000** |
| II:Pbi-Esophagus:Bik | -216.92 | -254.39 | -179.45 | **0.000** |
| IM:Pbi-Esophagus:Bik | -219.69 | -257.15 | -182.22 | **0.000** |
| Esophagus:Pgy-Esophagus:Bik | -51.79 | -89.25 | -14.32 | **0.001** |
| IF:Pgy-Esophagus:Bik | -211.76 | -249.22 | -174.29 | **0.000** |
| II:Pgy-Esophagus:Bik | -197.21 | -234.68 | -159.74 | **0.000** |
| IM:Pgy-Esophagus:Bik | -204.06 | -241.53 | -166.59 | **0.000** |
| II:Bik-IF:Bik | -15.15 | -52.62 | 22.32 | 0.960 |
| IM:Bik-IF:Bik | -28.89 | -66.36 | 8.57 | 0.283 |
| Esophagus:Pbi-IF:Bik | 236.44 | 198.98 | 273.91 | **0.000** |
| IF:Pbi-IF:Bik | -27.69 | -65.15 | 9.78 | 0.343 |
| II:Pbi-IF:Bik | -18.90 | -56.37 | 18.57 | 0.844 |
| IM:Pbi-IF:Bik | -21.67 | -59.13 | 15.80 | 0.700 |
| Esophagus:Pgy-IF:Bik | 146.23 | 108.77 | 183.70 | **0.000** |
| IF:Pgy-IF:Bik | -13.74 | -51.20 | 23.73 | 0.980 |
| II:Pgy-IF:Bik | 0.81 | -36.65 | 38.28 | 1.000 |
| IM:Pgy-IF:Bik | -6.04 | -43.51 | 31.43 | 1.000 |
| IM:Bik-II:Bik | -13.74 | -51.21 | 23.72 | 0.980 |
| Esophagus:Pbi-II:Bik | 251.59 | 214.13 | 289.06 | **0.000** |
| IF:Pbi-II:Bik | -12.54 | -50.00 | 24.93 | 0.990 |
| II:Pbi-II:Bik | -3.75 | -41.22 | 33.72 | 1.000 |
| IM:Pbi-II:Bik | -6.52 | -43.98 | 30.95 | 1.000 |
| Esophagus:Pgy-II:Bik | 161.38 | 123.92 | 198.85 | **0.000** |
| IF:Pgy-II:Bik | 1.41 | -36.05 | 38.88 | 1.000 |
| II:Pgy-II:Bik | 15.96 | -21.50 | 53.43 | 0.943 |
| IM:Pgy-II:Bik | 9.11 | -28.36 | 46.58 | 0.999 |
| Esophagus:Pbi-IM:Bik | 265.34 | 227.87 | 302.80 | **0.000** |
| IF:Pbi-IM:Bik | 1.21 | -36.26 | 38.67 | 1.000 |
| II:Pbi-IM:Bik | 9.99 | -27.47 | 47.46 | 0.999 |
| IM:Pbi-IM:Bik | 7.23 | -30.24 | 44.69 | 1.000 |
| Esophagus:Pgy-IM:Bik | 175.13 | 137.66 | 212.59 | **0.000** |
| IF:Pgy-IM:Bik | 15.16 | -22.31 | 52.62 | 0.960 |
| II:Pgy-IM:Bik | 29.70 | -7.76 | 67.17 | 0.247 |
| IM:Pgy-IM:Bik | 22.85 | -14.61 | 60.32 | 0.629 |
| IF:Pbi-Esophagus:Pbi | -264.13 | -301.60 | -226.66 | **0.000** |
| II:Pbi-Esophagus:Pbi | -255.35 | -292.81 | -217.88 | **0.000** |
| IM:Pbi-Esophagus:Pbi | -258.11 | -295.58 | -220.64 | **0.000** |
| Esophagus:Pgy-Esophagus:Pbi | -90.21 | -127.68 | -52.74 | **0.000** |
| IF:Pgy-Esophagus:Pbi | -250.18 | -287.65 | -212.71 | **0.000** |
| II:Pgy-Esophagus:Pbi | -235.63 | -273.10 | -198.17 | **0.000** |
| IM:Pgy-Esophagus:Pbi | -242.49 | -279.95 | -205.02 | **0.000** |
| II:Pbi-IF:Pbi | 8.79 | -28.68 | 46.25 | 1.000 |
| IM:Pbi-IF:Pbi | 6.02 | -31.45 | 43.49 | 1.000 |
| Esophagus:Pgy-IF:Pbi | 173.92 | 136.45 | 211.39 | **0.000** |
| IF:Pgy-IF:Pbi | 13.95 | -23.52 | 51.42 | 0.978 |
| II:Pgy-IF:Pbi | 28.50 | -8.97 | 65.96 | 0.302 |
| IM:Pgy-IF:Pbi | 21.65 | -15.82 | 59.11 | 0.702 |
| IM:Pbi-II:Pbi | -2.77 | -40.23 | 34.70 | 1.000 |
| Esophagus:Pgy-II:Pbi | 165.13 | 127.67 | 202.60 | **0.000** |
| IF:Pgy-II:Pbi | 5.17 | -32.30 | 42.63 | 1.000 |
| II:Pgy-II:Pbi | 19.71 | -17.75 | 57.18 | 0.806 |
| IM:Pgy-II:Pbi | 12.86 | -24.61 | 50.33 | 0.988 |
| Esophagus:Pgy-IM:Pbi | 167.90 | 130.43 | 205.37 | **0.000** |
| IF:Pgy-IM:Pbi | 7.93 | -29.54 | 45.40 | 1.000 |
| II:Pgy-IM:Pbi | 22.48 | -14.99 | 59.94 | 0.652 |
| IM:Pgy-IM:Pbi | 15.63 | -21.84 | 53.09 | 0.951 |
| IF:Pgy-Esophagus:Pgy | -159.97 | -197.44 | -122.50 | **0.000** |
| II:Pgy-Esophagus:Pgy | -145.42 | -182.89 | -107.96 | **0.000** |
| IM:Pgy-Esophagus:Pgy | -152.27 | -189.74 | -114.81 | **0.000** |
| II:Pgy-IF:Pgy | 14.55 | -22.92 | 52.01 | 0.970 |
| IM:Pgy-IF:Pgy | 7.69 | -29.77 | 45.16 | 1.000 |
| IM:Pgy-II:Pgy | -6.85 | -44.32 | 30.61 | 1.000 |

**Table S4** – *Post hoc* of Analysis of Variance (ANOVA) applied to lumen perimeter of digestive tract of omnivorous tetra fishes from neotropical streams, southern Brazil. Significant p-values are in bold.

| Segment/Species | diff | lwr | upr | p adj |
| --- | --- | --- | --- | --- |
| IF:Bik-Esophagus:Bik | 2606.14 | -14342.03 | 19554.32 | 1.000 |
| II:Bik-Esophagus:Bik | 1828.65 | -15119.52 | 18776.82 | 1.000 |
| IM:Bik-Esophagus:Bik | -3719.18 | -20667.35 | 13229.00 | 1.000 |
| Esophagus:Pbi-Esophagus:Bik | 1430.24 | -15517.94 | 18378.41 | 1.000 |
| IF:Pbi-Esophagus:Bik | 18577.03 | 1628.86 | 35525.20 | **0.021** |
| II:Pbi-Esophagus:Bik | 3953.11 | -12995.06 | 20901.29 | 1.000 |
| IM:Pbi-Esophagus:Bik | -914.65 | -17862.82 | 16033.52 | 1.000 |
| Esophagus:Pgy-Esophagus:Bik | 262.09 | -16686.08 | 17210.26 | 1.000 |
| IF:Pgy-Esophagus:Bik | 19431.80 | 2483.63 | 36379.97 | **0.013** |
| II:Pgy-Esophagus:Bik | 20090.75 | 3142.58 | 37038.93 | **0.009** |
| IM:Pgy-Esophagus:Bik | 19103.14 | 2154.97 | 36051.32 | **0.015** |
| II:Bik-IF:Bik | -777.49 | -17725.67 | 16170.68 | 1.000 |
| IM:Bik-IF:Bik | -6325.32 | -23273.49 | 10622.86 | 0.978 |
| Esophagus:Pbi-IF:Bik | -1175.91 | -18124.08 | 15772.27 | 1.000 |
| IF:Pbi-IF:Bik | 15970.89 | -977.29 | 32919.06 | 0.082 |
| II:Pbi-IF:Bik | 1346.97 | -15601.20 | 18295.14 | 1.000 |
| IM:Pbi-IF:Bik | -3520.79 | -20468.97 | 13427.38 | 1.000 |
| Esophagus:Pgy-IF:Bik | -2344.05 | -19292.23 | 14604.12 | 1.000 |
| IF:Pgy-IF:Bik | 16825.66 | -122.52 | 33773.83 | 0.053 |
| II:Pgy-IF:Bik | 17484.61 | 536.44 | 34432.78 | **0.038** |
| IM:Pgy-IF:Bik | 16497.00 | -451.17 | 33445.17 | 0.063 |
| IM:Bik-II:Bik | -5547.83 | -22496.00 | 11400.35 | 0.992 |
| Esophagus:Pbi-II:Bik | -398.41 | -17346.59 | 16549.76 | 1.000 |
| IF:Pbi-II:Bik | 16748.38 | -199.79 | 33696.55 | 0.055 |
| II:Pbi-II:Bik | 2124.46 | -14823.71 | 19072.64 | 1.000 |
| IM:Pbi-II:Bik | -2743.30 | -19691.47 | 14204.87 | 1.000 |
| Esophagus:Pgy-II:Bik | -1566.56 | -18514.73 | 15381.61 | 1.000 |
| IF:Pgy-II:Bik | 17603.15 | 654.98 | 34551.32 | **0.035** |
| II:Pgy-II:Bik | 18262.10 | 1313.93 | 35210.28 | **0.025** |
| IM:Pgy-II:Bik | 17274.49 | 326.32 | 34222.67 | **0.042** |
| Esophagus:Pbi-IM:Bik | 5149.41 | -11798.76 | 22097.59 | 0.996 |
| IF:Pbi-IM:Bik | 22296.20 | 5348.03 | 39244.38 | **0.002** |
| II:Pbi-IM:Bik | 7672.29 | -9275.88 | 24620.46 | 0.917 |
| IM:Pbi-IM:Bik | 2804.53 | -14143.65 | 19752.70 | 1.000 |
| Esophagus:Pgy-IM:Bik | 3981.27 | -12966.91 | 20929.44 | 1.000 |
| IF:Pgy-IM:Bik | 23150.98 | 6202.80 | 40099.15 | **0.001** |
| II:Pgy-IM:Bik | 23809.93 | 6861.76 | 40758.10 | **0.001** |
| IM:Pgy-IM:Bik | 22822.32 | 5874.15 | 39770.49 | **0.002** |
| IF:Pbi-Esophagus:Pbi | 17146.79 | 198.62 | 34094.97 | **0.045** |
| II:Pbi-Esophagus:Pbi | 2522.88 | -14425.30 | 19471.05 | 1.000 |
| IM:Pbi-Esophagus:Pbi | -2344.89 | -19293.06 | 14603.29 | 1.000 |
| Esophagus:Pgy-Esophagus:Pbi | -1168.15 | -18116.32 | 15780.03 | 1.000 |
| IF:Pgy-Esophagus:Pbi | 18001.56 | 1053.39 | 34949.74 | **0.028** |
| II:Pgy-Esophagus:Pbi | 18660.52 | 1712.35 | 35608.69 | **0.020** |
| IM:Pgy-Esophagus:Pbi | 17672.91 | 724.73 | 34621.08 | **0.034** |
| II:Pbi-IF:Pbi | -14623.92 | -31572.09 | 2324.26 | 0.152 |
| IM:Pbi-IF:Pbi | -19491.68 | -36439.85 | -2543.51 | **0.012** |
| Esophagus:Pgy-IF:Pbi | -18314.94 | -35263.11 | -1366.77 | **0.024** |
| IF:Pgy-IF:Pbi | 854.77 | -16093.40 | 17802.94 | 1.000 |
| II:Pgy-IF:Pbi | 1513.72 | -15434.45 | 18461.90 | 1.000 |
| IM:Pgy-IF:Pbi | 526.11 | -16422.06 | 17474.29 | 1.000 |
| IM:Pbi-II:Pbi | -4867.76 | -21815.94 | 12080.41 | 0.997 |
| Esophagus:Pgy-II:Pbi | -3691.02 | -20639.20 | 13257.15 | 1.000 |
| IF:Pgy-II:Pbi | 15478.69 | -1469.49 | 32426.86 | 0.103 |
| II:Pgy-II:Pbi | 16137.64 | -810.53 | 33085.81 | 0.075 |
| IM:Pgy-II:Pbi | 15150.03 | -1798.14 | 32098.20 | 0.120 |
| Esophagus:Pgy-IM:Pbi | 1176.74 | -15771.43 | 18124.91 | 1.000 |
| IF:Pgy-IM:Pbi | 20346.45 | 3398.28 | 37294.62 | **0.007** |
| II:Pgy-IM:Pbi | 21005.40 | 4057.23 | 37953.58 | **0.005** |
| IM:Pgy-IM:Pbi | 20017.79 | 3069.62 | 36965.97 | **0.009** |
| IF:Pgy-Esophagus:Pgy | 19169.71 | 2221.54 | 36117.88 | **0.015** |
| II:Pgy-Esophagus:Pgy | 19828.66 | 2880.49 | 36776.84 | **0.010** |
| IM:Pgy-Esophagus:Pgy | 18841.05 | 1892.88 | 35789.23 | **0.018** |
| II:Pgy-IF:Pgy | 658.95 | -16289.22 | 17607.13 | 1.000 |
| IM:Pgy-IF:Pgy | -328.66 | -17276.83 | 16619.52 | 1.000 |
| IM:Pgy-II:Pgy | -987.61 | -17935.78 | 15960.56 | 1.000 |

**Table S5** – *Post hoc* of Analysis of Variance (ANOVA) applied to lumen area of digestive tract of omnivorous tetra fishes from neotropical streams, southern Brazil. Significant p-values are in bold.

| Segment/Species | diff | lwr | upr | p adj |
| --- | --- | --- | --- | --- |
| IF:Bik-Esophagus:Bik | -319625.84 | -1415502.32 | 776250.63 | 0.997 |
| II:Bik-Esophagus:Bik | 992379.35 | -103497.13 | 2088255.83 | 0.110 |
| IM:Bik-Esophagus:Bik | 269414.14 | -826462.34 | 1365290.62 | 0.999 |
| Esophagus:Pbi-Esophagus:Bik | -194355.97 | -1290232.44 | 901520.51 | 1.000 |
| IF:Pbi-Esophagus:Bik | 969137.35 | -126739.13 | 2065013.83 | 0.129 |
| II:Pbi-Esophagus:Bik | 672201.80 | -423674.68 | 1768078.28 | 0.621 |
| IM:Pbi-Esophagus:Bik | 498372.90 | -597503.58 | 1594249.38 | 0.914 |
| Esophagus:Pgy-Esophagus:Bik | -305004.67 | -1400881.15 | 790871.81 | 0.998 |
| IF:Pgy-Esophagus:Bik | 184037.32 | -911839.15 | 1279913.80 | 1.000 |
| II:Pgy-Esophagus:Bik | 249515.81 | -846360.67 | 1345392.29 | 1.000 |
| IM:Pgy-Esophagus:Bik | 447647.33 | -648229.15 | 1543523.80 | 0.957 |
| II:Bik-IF:Bik | 1312005.20 | 216128.72 | 2407881.67 | **0.008** |
| IM:Bik-IF:Bik | 589039.99 | -506836.49 | 1684916.46 | 0.785 |
| Esophagus:Pbi-IF:Bik | 125269.88 | -970606.60 | 1221146.36 | 1.000 |
| IF:Pbi-IF:Bik | 1288763.20 | 192886.72 | 2384639.67 | **0.009** |
| II:Pbi-IF:Bik | 991827.64 | -104048.83 | 2087704.12 | 0.110 |
| IM:Pbi-IF:Bik | 817998.74 | -277877.73 | 1913875.22 | 0.328 |
| Esophagus:Pgy-IF:Bik | 14621.17 | -1081255.30 | 1110497.65 | 1.000 |
| IF:Pgy-IF:Bik | 503663.17 | -592213.31 | 1599539.65 | 0.909 |
| II:Pgy-IF:Bik | 569141.65 | -526734.83 | 1665018.13 | 0.819 |
| IM:Pgy-IF:Bik | 767273.17 | -328603.31 | 1863149.65 | 0.423 |
| IM:Bik-II:Bik | -722965.21 | -1818841.69 | 372911.27 | 0.514 |
| Esophagus:Pbi-II:Bik | -1186735.32 | -2282611.80 | -90858.84 | **0.023** |
| IF:Pbi-II:Bik | -23242.00 | -1119118.48 | 1072634.48 | 1.000 |
| II:Pbi-II:Bik | -320177.55 | -1416054.03 | 775698.93 | 0.997 |
| IM:Pbi-II:Bik | -494006.45 | -1589882.93 | 601870.03 | 0.919 |
| Esophagus:Pgy-II:Bik | -1297384.02 | -2393260.50 | -201507.54 | **0.009** |
| IF:Pgy-II:Bik | -808342.03 | -1904218.51 | 287534.45 | 0.345 |
| II:Pgy-II:Bik | -742863.54 | -1838740.02 | 353012.94 | 0.472 |
| IM:Pgy-II:Bik | -544732.03 | -1640608.50 | 551144.45 | 0.856 |
| Esophagus:Pbi-IM:Bik | -463770.11 | -1559646.58 | 632106.37 | 0.946 |
| IF:Pbi-IM:Bik | 699723.21 | -396153.27 | 1795599.69 | 0.563 |
| II:Pbi-IM:Bik | 402787.66 | -693088.82 | 1498664.14 | 0.980 |
| IM:Pbi-IM:Bik | 228958.76 | -866917.72 | 1324835.24 | 1.000 |
| Esophagus:Pgy-IM:Bik | -574418.81 | -1670295.29 | 521457.67 | 0.810 |
| IF:Pgy-IM:Bik | -85376.82 | -1181253.30 | 1010499.66 | 1.000 |
| II:Pgy-IM:Bik | -19898.33 | -1115774.81 | 1075978.15 | 1.000 |
| IM:Pgy-IM:Bik | 178233.18 | -917643.29 | 1274109.66 | 1.000 |
| IF:Pbi-Esophagus:Pbi | 1163493.32 | 67616.84 | 2259369.79 | **0.029** |
| II:Pbi-Esophagus:Pbi | 866557.76 | -229318.71 | 1962434.24 | 0.250 |
| IM:Pbi-Esophagus:Pbi | 692728.86 | -403147.61 | 1788605.34 | 0.578 |
| Esophagus:Pgy-Esophagus:Pbi | -110648.71 | -1206525.18 | 985227.77 | 1.000 |
| IF:Pgy-Esophagus:Pbi | 378393.29 | -717483.19 | 1474269.77 | 0.988 |
| II:Pgy-Esophagus:Pbi | 443871.77 | -652004.70 | 1539748.25 | 0.960 |
| IM:Pgy-Esophagus:Pbi | 642003.29 | -453873.19 | 1737879.77 | 0.684 |
| II:Pbi-IF:Pbi | -296935.55 | -1392812.03 | 798940.93 | 0.998 |
| IM:Pbi-IF:Pbi | -470764.45 | -1566640.93 | 625112.03 | 0.940 |
| Esophagus:Pgy-IF:Pbi | -1274142.02 | -2370018.50 | -178265.54 | **0.011** |
| IF:Pgy-IF:Pbi | -785100.03 | -1880976.50 | 310776.45 | 0.388 |
| II:Pgy-IF:Pbi | -719621.54 | -1815498.02 | 376254.94 | 0.521 |
| IM:Pgy-IF:Pbi | -521490.02 | -1617366.50 | 574386.45 | 0.888 |
| IM:Pbi-II:Pbi | -173828.90 | -1269705.38 | 922047.58 | 1.000 |
| Esophagus:Pgy-II:Pbi | -977206.47 | -2073082.95 | 118670.01 | 0.122 |
| IF:Pgy-II:Pbi | -488164.48 | -1584040.95 | 607712.00 | 0.925 |
| II:Pgy-II:Pbi | -422685.99 | -1518562.47 | 673190.49 | 0.972 |
| IM:Pgy-II:Pbi | -224554.47 | -1320430.95 | 871322.00 | 1.000 |
| Esophagus:Pgy-IM:Pbi | -803377.57 | -1899254.05 | 292498.91 | 0.354 |
| IF:Pgy-IM:Pbi | -314335.57 | -1410212.05 | 781540.90 | 0.997 |
| II:Pgy-IM:Pbi | -248857.09 | -1344733.57 | 847019.39 | 1.000 |
| IM:Pgy-IM:Pbi | -50725.57 | -1146602.05 | 1045150.91 | 1.000 |
| IF:Pgy-Esophagus:Pgy | 489042.00 | -606834.48 | 1584918.47 | 0.924 |
| II:Pgy-Esophagus:Pgy | 554520.48 | -541356.00 | 1650396.96 | 0.842 |
| IM:Pgy-Esophagus:Pgy | 752652.00 | -343224.48 | 1848528.47 | 0.452 |
| II:Pgy-IF:Pgy | 65478.48 | -1030397.99 | 1161354.96 | 1.000 |
| IM:Pgy-IF:Pgy | 263610.00 | -832266.48 | 1359486.48 | 0.999 |
| IM:Pgy-II:Pgy | 198131.52 | -897744.96 | 1294008.00 | 1.000 |

**Table S6** – *Post hoc* of Analysis of Variance (ANOVA) applied to the number of goblet cells in different segments of the intestine of omnivorous tetra fishes from neotropical streams, southern Brazil. Significant p-values are in bold.

| Segment | diff | lwr | upr | p adj |
| --- | --- | --- | --- | --- |
| II-IF | -34.60 | -38.13 | -31.07 | **0.000** |
| IM-IF | -31.60 | -35.13 | -28.07 | **0.000** |
| IM-II | 3.00 | -0.53 | 6.53 | 0.109 |

**Table S7.** Summary of stable isotope values for animal resources, plant resources, SOM and omnivorous fish in Neotropical headwater streams, southern Brazil, used in isotope mixing models.

| Stream | Taxon | n | δ^13^C (mean ± SD) | δ^15^N (mean ± SD) |
| --- | --- | --- | --- | --- |
| S1 | Pbi | 15 | -23.70 ± 0.95 | 9.64 ± 0.46 |
|  | Pgy | 15 | -24.34 ± 0.55 | 9.90 ± 0.51 |
|  | Animal resources | 12 | -23.53 ± 2.04 | 7.03 ± 0.78 |
|  | Plants resources | 3 | -31.92 ± 0.56 | -1.80 ± 0.72 |
|  | SOM | 1 | -22.74 | 5.63 |
| S2 | Pbi | 15 | -25.84 ± 0.95 | 9.65 ± 1.32 |
|  | Bik | 10 | -25.31 ± 0.94 | 9.34 ± 0.75 |
|  | Animal resources | 11 | -26.53 ± 4.59 | 5.18 ± 1.76 |
|  | Plants resources | 3 | -33.45 ± 0.49 | 0.13 ± 0.33 |
|  | SOM | 1 | -26.93 | 4.12 |

**Table S8** – Summary of relative isotopic contribution of food resources to the diet of omnivorous tetra fishes from neotropical streams, estimated by a MixSIAR Bayesian mixing model. Epsilon: multiplicative error term (if model uses “Residual * Process” error).

| Site |  |  | Mean | SD | 2.50% | 5% | 25% | 50% | 75% | 95% | 97.50% |
| --- | --- | --- | --- | --- | --- | --- | --- | --- | --- | --- | --- |
| S1 | Epsilon.1 |  | 1.23 | 1.94 | 0.06 | 0.09 | 0.19 | 0.43 | 1.37 | 5.11 | 6.86 |
|  | Epsilon.2 |  | 0.35 | 0.34 | 0.02 | 0.03 | 0.10 | 0.25 | 0.49 | 0.97 | 1.24 |
|  | Pbi | Animal | 0.59 | 0.28 | 0.04 | 0.10 | 0.34 | 0.66 | 0.83 | 0.94 | 0.96 |
|  | Pgy |  | 0.52 | 0.35 | 0.02 | 0.03 | 0.16 | 0.54 | 0.87 | 0.97 | 0.98 |
|  | Pbi | Plants | 0.17 | 0.12 | 0.01 | 0.01 | 0.05 | 0.17 | 0.28 | 0.35 | 0.37 |
|  | Pgy |  | 0.21 | 0.16 | 0.00 | 0.00 | 0.04 | 0.24 | 0.35 | 0.43 | 0.45 |
|  | Pbi | SOM | 0.24 | 0.20 | 0.01 | 0.01 | 0.08 | 0.20 | 0.39 | 0.60 | 0.65 |
|  | Pgy |  | 0.28 | 0.23 | 0.00 | 0.01 | 0.05 | 0.21 | 0.50 | 0.64 | 0.68 |
| S2 | Epsilon.1 |  | 6.36 | 4.74 | 0.09 | 0.15 | 2.05 | 6.14 | 9.66 | 14.91 | 16.67 |
|  | Epsilon.2 |  | 0.35 | 0.30 | 0.03 | 0.05 | 0.15 | 0.27 | 0.47 | 0.95 | 1.12 |
|  | Pbi | Animal | 0.51 | 0.17 | 0.00 | 0.00 | 0.02 | 0.04 | 0.10 | 0.50 | 0.70 |
|  | Bik |  | 0.51 | 0.19 | 0.00 | 0.00 | 0.01 | 0.03 | 0.11 | 0.57 | 0.77 |
|  | Pbi | Plants | 0.07 | 0.07 | 0.00 | 0.01 | 0.02 | 0.05 | 0.10 | 0.20 | 0.24 |
|  | Bik |  | 0.04 | 0.06 | 0.00 | 0.00 | 0.01 | 0.02 | 0.06 | 0.15 | 0.20 |
|  | Pbi | SOM | 0.42 | 0.19 | 0.18 | 0.36 | 0.80 | 0.89 | 0.94 | 0.98 | 0.98 |
|  | Bik |  | 0.45 | 0.21 | 0.15 | 0.33 | 0.83 | 0.93 | 0.97 | 0.99 | 1.00 |
